# Muscle symptoms and a latent physiological symptom-dysregulation factor in ME/CFS: exploratory analyses by sex and age-defined menopausal-status proxy group

**DOI:** 10.1186/s12967-026-09018-9

**Published:** 2026-09-28

**Authors:** Lotte Habermann-Horstmeier, Lukas M. Horstmeier

**Affiliations:** 1 Villingen Institute of Public Health (VIPH), Klosterring 5, D-78050 Villingen-Schwenningen, Germany; 2 Institute for Medical Biometry and Statistics, University Hospital Freiburg, Section for Health Services Research and Rehabilitation Research (SEVERA), Hugstetter Str. 49, D-79106 Freiburg, Germany

**Keywords:** Myalgic encephalomyelitis/chronic fatigue syndrome (ME/CFS), Post-exertional malaise, Muscle symptoms, Physiological dysregulation, Sex differences

## Abstract

**Background:**

Muscle-related symptoms are among the most disabling manifestations of myalgic encephalomyelitis/chronic fatigue syndrome (ME/CFS), yet their position within the broader symptom architecture of the disease remains unclear. We hypothesized that muscle symptoms may represent a candidate integrative symptom within a broader shared symptom-dysregulation structure and their symptom associations differ according to sex and menopausal status.

**Methods:**

Cross-sectional data from 736 individuals with physician-diagnosed ME/CFS enrolled in the APAV-ME/CFS registry were analysed. Muscle problems served as the primary outcome variable. Associations with 14 symptom domains were examined using multivariable logistic regression. Pearson and tetrachoric correlations, exploratory factor analysis, and structural equation modeling (SEM) were used to identify latent symptom structures. Exploratory analyses were stratified by sex, menopausal status, and disease duration.

**Results:**

In the full multivariable model (non-robust estimates), breathing problems (OR 2.49 [95% Conf. Interval: 1.42–4.38], *p* = 0.001), flu-like symptoms (OR 1.97 [1.14–3.42], *p* = 0.015), and temperature-regulation disorders (OR 1.97 [1.10–3.51], *p* = 0.021) independently predicted muscle problems. A reduced physiological model (not-robust estimates) additionally identified cardiovascular symptoms (OR 1.87 [1.09–3.24], *p* = 0.024) as a significant predictor. Tetrachoric correlations demonstrated substantial latent associations between muscle problems and breathing difficulties (ρ = 0.52), cardiovascular symptoms (ρ = 0.49), temperature-regulation disorders (ρ = 0.49), visual disturbances (ρ = 0.39), and flu-like symptoms (ρ = 0.39). Factor analysis and SEM supported a single latent physiological dysregulation factor with good model fit (RMSEA = 0.046, CFI = 0.971, TLI = 0.952, SRMR = 0.026). The sex-stratified analyses revealed differences in the pattern of statistically significant symptom associations across the subgroups. However, the overall test of the symptom-by-sex interaction terms did not provide evidence for a statistically established difference in the symptom associations by sex. Thus, the subgroup-specific patterns should primarily be interpreted as descriptive and exploratory. At the individual symptom level, the association between breathing-related symptoms and muscle problems showed a statistically significant interaction with sex, suggesting that this association may differ between women and men. However, this finding should be interpreted cautiously given the non-significant overall interaction test and the wide confidence interval of the interaction estimate.

In women, all five physiological symptoms independently predicted muscle problems, whereas in men only breathing problems and temperature-regulation disorders remained significant. Women classified as premenopausal and postmenopausal based on an age-defined proxy showed different patterns of symptom associations with muscle problems in subgroup-specific analyses. Women in the premenopausal age-proxy group showed significant associations with cardiovascular symptoms, visual disturbances, and temperature-regulation disorders, whereas women classified as postmenopausal showed significant associations with breathing difficulties and flu-like symptoms. However, the overall menopausal-by-symptom interaction was not statistically significant (Wald χ^2^ (5)=5.96, *p* = 0.310).

The specified latent physiological factor showed statistical comparability across the cross-sectional disease-duration groups. Gastrointestinal complaints loaded substantially on the latent factor despite lacking an independent association with muscle problems in multivariable regression. Urogenital symptoms also showed a smaller but significant association with the latent factor and substantial item-specific variance.

**Conclusions:**

Muscle symptoms may represent a candidate integrative symptom within a latent physiological symptom-dysregulation factor encompassing respiratory, cardiovascular, thermoregulatory, visual, and flu-like symptoms in this ME/CFS sample. Secondary analyses suggested that gastrointestinal complaints were also part of the broader physiological symptom pattern, although they were not independently associated with muscle problems. The specified latent physiological factor showed no overall statistical evidence of measurement differences across the cross-sectional disease-duration groups. However, subgroup-specific symptom patterns differed descriptively by sex and age-defined menopausal-status proxy group. Women classified as premenopausal were characterized primarily by cardiovascular, visual, and thermoregulatory symptom associations, whereas women classified as postmenopausal and men showed greater prominence of breathing-related symptoms. Flu-like symptoms were independently associated with muscle problems in the postmenopausal subgroup but not in the premenopausal subgroup. Because the corresponding interaction term was not statistically significant, this subgroup contrast should be interpreted as an exploratory difference in association rather than as evidence of a menopause-specific effect.

More broadly, the findings are compatible with the hypothesis that the latent symptom-dysregulation factor may reflect a chronic PEM-associated multisystem response. Whether this symptom-dysregulation factor corresponds to interacting autonomic, vascular, immunological, and metabolic processes remains to be determined by longitudinal and biomarker-based studies. ME/CFS phenotyping may improve patient stratification and facilitate the development of mechanism-based therapeutic approaches.

**Graphical Abstract:**

**Figure:**
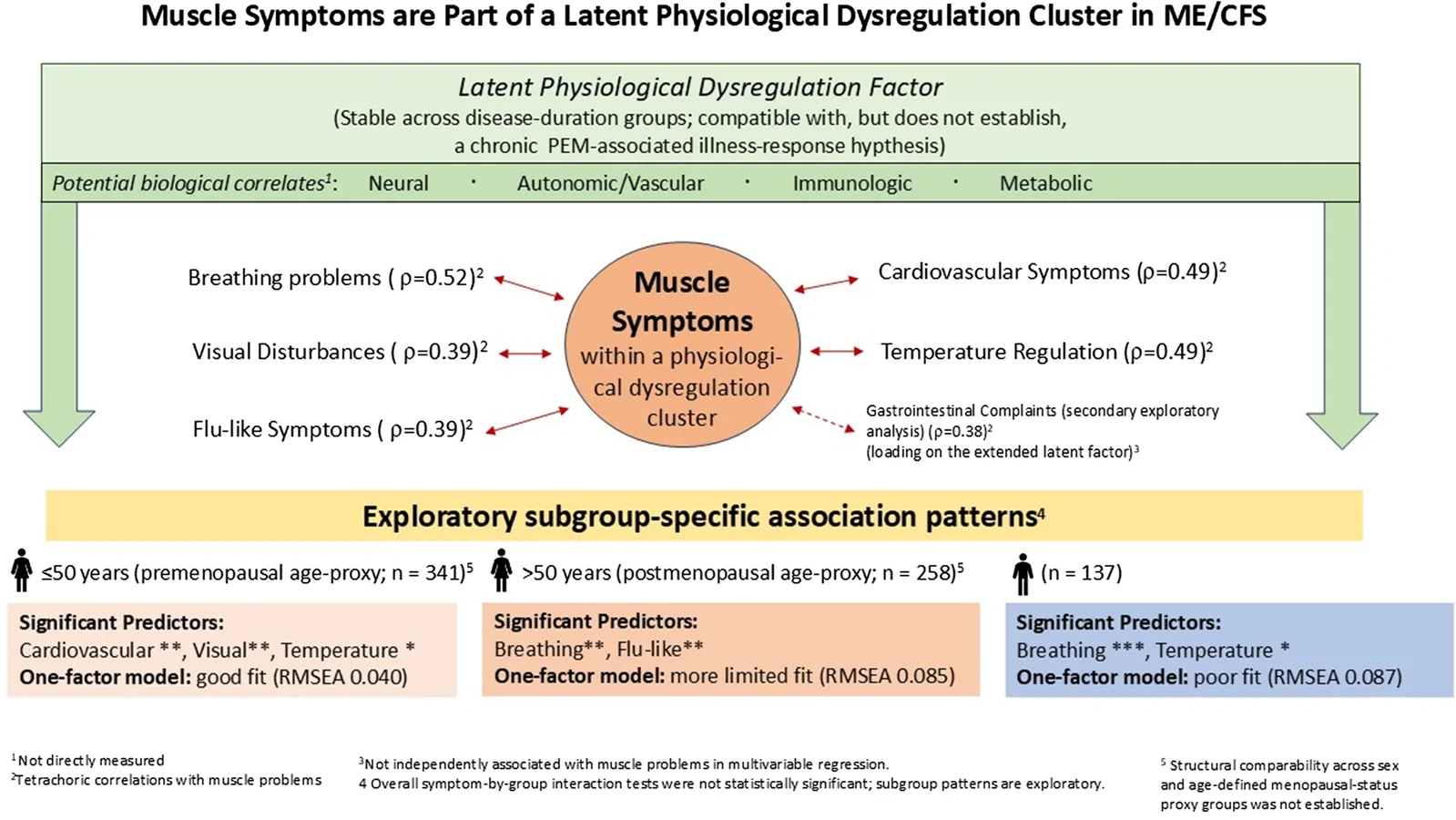

**Supplementary information:**

The online version contains supplementary material available at https://doi.org/10.1186/s12967-026-09018-9.

## Introduction

Muscle-related symptoms are among the most frequent and disabling manifestations of myalgic encephalomyelitis/chronic fatigue syndrome (ME/CFS), yet their pathophysiological basis remains incompletely understood. Patients commonly report exertional muscle pain, very early fatigability, muscle twitching and cramps, tremor, heaviness—even to the point of a complete inability to perform movements—as well as severely delayed recovery following even minimal physical activity [1–3]. These symptoms contribute substantially to functional impairment and are closely linked to post-exertional malaise (PEM), the cardinal feature of ME/CFS. Previous studies have consistently demonstrated that musculoskeletal pain and exercise-induced symptom exacerbation represent major determinants of disease burden in affected individuals [1, 4–6].

Despite their clinical relevance, attempts to attribute muscle symptoms to a single biological mechanism have been largely unsuccessful. Instead, symptom-based investigations have identified heterogeneous and partially overlapping symptom domains involving autonomic dysfunction, flu-like symptoms, cognitive impairment, sleep disturbances, and respiratory complaints. These findings suggest that muscle symptoms may arise from multiple interacting physiological processes rather than from isolated skeletal muscle pathology alone [7, 8].

Several mechanistic domains have been implicated in the pathophysiology of ME/CFS, including persistent or intermittent immune activation, autonomic nervous system dysregulation, endothelial dysfunction, and abnormalities in cellular energy metabolism [9]. Impaired mitochondrial function, altered oxidative phosphorylation, reduced oxygen extraction, and abnormalities in neuromuscular performance have been reported [3], although findings have varied across patient cohorts and study methodologies. More recent evidence highlights disturbances in microcirculatory substrate delivery, nitric oxide-dependent vascular regulation, endothelial function, and autoantibodies targeting G-protein-coupled receptors (GPCRs), particularly β2-adrenergic and muscarinic receptors [4]. Together, these observations support the concept of a multisystem disorder in which impaired vascular and metabolic regulation may contribute substantially to muscle dysfunction and exercise intolerance [2, 6, 9].

Hormonal modulation adds a further layer of complexity. Estrogen influences vascular tone, endothelial function, immune signaling, and energy metabolism. Growing evidence suggests that symptom expression and disease trajectories differ between men and women. In this context, endocrine transitions such as menopause may alter symptom profiles and potentially influence the interaction between autonomic, immune, and vascular mechanisms in ME/CFS [10].

The present study builds on a series of analyses conducted within the APAV ME/CFS research program. In the first phase (Studies 1–2), we hypothesized that distinct symptom groups would emerge that correspond to established pathophysiological domains, particularly neuroimmune, autonomic, gastrointestinal, and central nervous system mechanisms. These analyses identified several biologically plausible symptom clusters that may facilitate improved patient stratification, support more precise diagnostic approaches, and inform the development of targeted therapeutic strategies [11, 12].

**Fig. 1:**
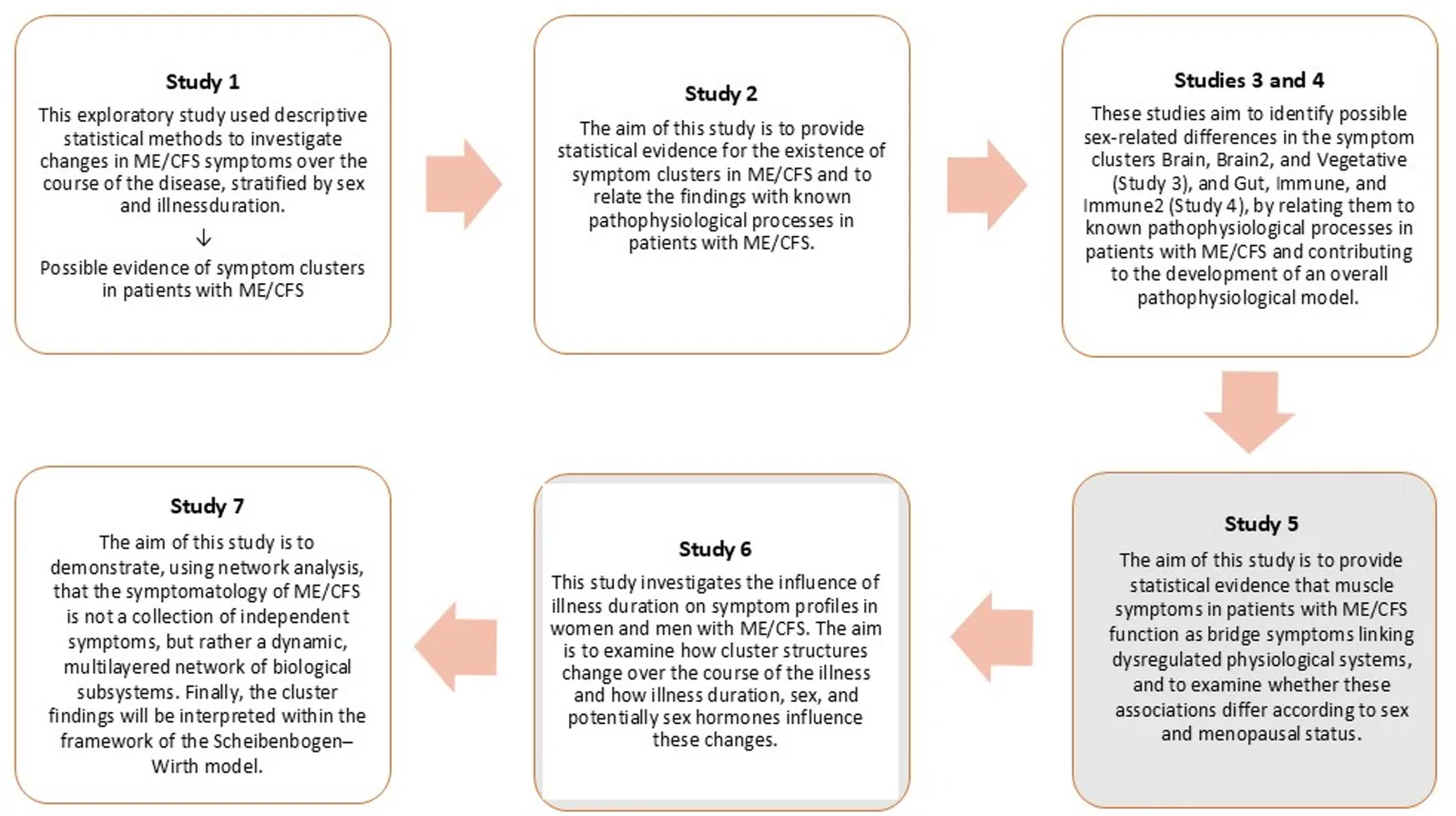
Chronological placement of study 5 within the sequence of studies

In the subsequent phase (Studies 3–4), we investigated potential modifiers of symptom architecture, focusing on biological sex and menopausal status. Study 3 examined sex-related differences in symptom clusters associated with autonomic and central nervous system dysfunction, whereas Study 4 focused on gastrointestinal and immune-related symptom domains. Together, these studies demonstrated substantial sex-specific variation in symptom organization and provided further support for an integrated pathophysiological model of ME/CFS [13, 14]. Now, the originally planned five-part study design was modified after the completion of Part 4. The results obtained thus far necessitated the expansion of the research program by two additional studies to ensure a comprehensive and systematic coverage of all relevant aspects. Accordingly, the final synthesis of the findings will be presented in Part 7 rather than in the originally envisaged Part 5 (see Fig. 1).

Against this background, the present study addresses a remaining question that emerged from the previous analyses: the role of muscle symptoms within the overall symptom architecture of ME/CFS. Although muscle complaints represent some of the most disabling manifestations of the disease, they did not consistently emerge as an independent symptom domain in earlier clustering analyses. This observation raises the possibility that muscle symptoms function as candidate integrative or connecting physiological symptoms linking multiple dysregulated systems—including autonomic, vascular, immune, and metabolic pathways—rather than constituting a distinct pathophysiological entity. Such an interpretation would be consistent with contemporary models emphasizing systemic dysregulation and impaired physiological integration in ME/CFS [1, 4].

The present study therefore complements the previous phases of the research program by specifically examining the position of muscle symptoms within the established symptom network and by exploring sex- and menopause-related differences in their manifestation.

The three objectives of this study (Study 5) are therefore:

1. To identify a physiological muscle symptom cluster within the APAV-ME/CFS dataset and determine whether muscle symptoms may represent a candidate integrative symptom within the broader symptom architecture.
2. To analyse sex- and menopause-specific differences in the structure and severity of muscle-related symptoms.
3. To integrate these findings into a mechanistic model linking symptom architecture with underlying autonomic, vascular, immunological, and metabolic pathways.

## Methods

### Study design and data source

This study represents the fifth phase of a series of symptom-architecture analyses conducted within the APAV-ME/CFS research program. The objective of the present analysis was to investigate the role of muscle-related symptoms within the broader symptom network of ME/CFS and to determine whether muscle symptoms form part of a distinct physiological dysregulation cluster. Particular emphasis was placed on sex- and menopause-related differences in symptom architecture.

The analyses were based on cross-sectional questionnaire data obtained from participants with physician-diagnosed ME/CFS who fulfilled the inclusion criteria of the APAV-ME/CFS registry. Details of recruitment procedures and the registry have been described previously [11, 12].

#### Symptom assessment and coding

Participants were asked to indicate the presence or absence of predefined ME/CFS-related symptoms or symptom groups based on symptom descriptions formulated with reference to the Canadian Consensus Criteria [15] and International Consensus Criteria [16]. Each symptom domain was assessed as a dichotomous variable (present/absent). The item “memory problems, word-finding difficulties, or brain fog” was coded as a single variable (Brainfog). Muscle problems comprised reported muscle pain, post-exertional muscle soreness, muscle twitching, or cramps. The remaining symptom domains were defined according to the symptom descriptions provided in Supplementary Table S1.

Of the 748 participants with physician-diagnosed ME/CFS, three participants reporting non-binary sex and nine participants with missing or incomplete questionnaire/sex data were not included in the present analyses. All analyses involving symptom variables were therefore restricted to these 736 participants. There were no missing responses within the completed symptom questionnaire.

#### Symptom variables

Participants reported the presence or absence of a broad range of ME/CFS-related symptoms. For the present study, muscle problems served as the primary variable of interest.

The following symptom domains were initially examined as potential correlates of muscle problems:

- Brain fog
- Headaches
- Visual disturbances
- Flu-like symptoms
- Susceptibility to infection
- Gastrointestinal complaints
- Urogenital complaints
- (Food) Intolerances
- Cardiovascular symptoms
- Breathing problems
- Sleep disturbances
- Sensory hypersensitivities
- Temperature-regulation disturbances
- Temperature intolerance

All variables were coded dichotomously (present/absent).

#### Sex and menopausal status

Sex was coded as female or male. The three individuals who identified as non-binary were excluded from the analysis prior to calculations due to the small group size. To investigate possible hormonal influences on symptom architecture, women were additionally stratified according to an age-defined proxy for menopausal status. Women aged 21–50 years were classified as the premenopausal proxy group, whereas women aged > 50 years were classified as the postmenopausal proxy group. This classification was based on the available age categories (21–25, 26–30, 31–40, 41–50, 51–60, 61–70, and >70 years) and therefore represents only a proxy for biological menopausal status. It does not capture perimenopausal fluctuations, individual variability in the timing of menopause, or the potential influence of hormone replacement therapy. Because detailed menopausal information was not collected, classification into pre- and postmenopausal groups remained approximate. Male participants (*n* = 137) were analysed separately under the sex-stratified analyses but were not included in menopause-specific models.

#### Disease duration

To explore the stability of the physiological symptom cluster over the course of illness, participants were stratified into three disease-duration categories:

- ≤5 years
- 6-10 years
- >10 years

Disease duration was available in predefined questionnaire categories. For the primary analysis, these categories were grouped as ≤5 years, 6–10 years, and >10 years to examine the statistical comparability of the specified latent symptom structure across the available cross-sectional disease-duration strata. In addition, to assess possible measurement invariance across disease duration, the data were divided into four disease-duration categories (≤2 years, 3–10 years, 11–20 years, and ≥21 years).

### Statistical analyses

All analyses were conducted using Stata 12.0.

#### Logistic regression

The primary analytic approach consisted of multivariable logistic regression models with muscle problems as the dependent variable. An initial full model included all symptom domains listed above. Based on the physiological rationale that muscle-related symptoms may reflect involvement of different muscle types, including skeletal, smooth, and cardiac muscle, a reduced physiological model was subsequently specified to examine symptom domains plausibly reflecting these muscle-related physiological systems involving different muscle types. The selection was guided by the predefined physiological symptom architecture and was not based solely on statistical significance in the full model. Therefore, the reduced model was a secondary analysis. It was not intended as a prespecified confirmatory model.

Separate logistic regression analyses were performed for:

- the total sample,
- women and men separately,
- women classified as premenopausal and women classified as postmenopausal,
- disease-duration subgroups.

To formally assess whether menopausal status modified the associations between the five physiological symptom domains and muscle problems, an interaction model was additionally fitted among women. The model included menopausal status, each of the five symptom domains and all menopausal-status-by-symptom interaction terms. Robust standard errors were used. The joint significance of the five interaction terms was assessed using a Wald test. Menopausal status was coded as 0 (=women classified as premenopausal, who were ≤ 50 years old) and 1 (women classified as postmenopausal, who were > 50 years old). The interaction terms therefore tested whether the log-odds associations between each symptom and muscle problems differed between the two menopausal-status groups. Statistical significance was defined as *p* < 0.05.

Given the exploratory nature and number of secondary and stratified analyses, the potential for inflated false-positive findings due to multiple comparisons was considered. The multivariable logistic regression including all 14 symptom domains was defined as the primary analysis addressing the prespecified primary research question. The reduced physiological model, sex-stratified analyses, age-defined menopausal-status analyses, disease-duration analyses, and the sensitivity analysis excluding women aged 41–50 years were considered secondary or exploratory analyses. No global multiplicity adjustment was applied because these analyses addressed distinct analytical questions and did not constitute a single prespecified family of hypothesis tests. Accordingly, findings from secondary and exploratory analyses were interpreted cautiously, with emphasis on effect estimates and 95% confidence intervals rather than statistical significance alone, and were not considered confirmatory solely on the basis of *p* < 0.05.

#### Correlation analyses

Because all symptom variables were dichotomous, both Pearson correlations and tetrachoric correlations were calculated. Pearson correlations were used to provide descriptive estimates of symptom co-occurrence. Tetrachoric correlations were used to estimate latent associations between symptom domains under the assumption that dichotomous symptom variables represent underlying continuous traits.

#### Exploratory factor analysis and structural equation modeling

Because all symptom domains were coded as binary variables (present/absent), associations among the symptom domains were initially examined using tetrachoric correlations. Exploratory factor analysis was then performed on the resulting tetrachoric correlation matrix using principal-factor extraction. The number of factors to retain was assessed using Horn’s parallel analysis with 100 random iterations and the 95th-percentile criterion. A factor was retained when its adjusted eigenvalue exceeded 1.

To examine whether the symptom domains could be represented by a common latent construct, a one-factor structural equation model (SEM) was subsequently specified, with muscle problems, flu-like symptoms, breathing problems, cardiovascular problems, temperature-regulation problems, and visual disturbances as indicators of a latent symptom-dysregulation factor. The SEM was estimated using maximum likelihood (ML) in Stata 12 without robust standard errors. The loading of muscle problems was fixed to 1.0 for model identification. Model fit was evaluated using the likelihood-ratio χ^2^ test, RMSEA with its 90% confidence interval, CFI, TLI, and SRMR. No threshold parameters were estimated in the SEM. Scalar invariance refers to equality of intercepts within the specified maximum-likelihood SEM treating the binary symptom indicators as observed 0/1 variables.

#### Factor structure of physiological symptoms

To evaluate whether the six physiological symptom domains could be represented by a common latent dimension, we examined their tetrachoric correlation matrix and conducted Horn’s parallel analysis. Because the symptom indicators were binary, tetrachoric correlations were used to account for their dichotomous nature. Parallel analysis was based on 100 random iterations and the 95th-percentile criterion. A one-factor solution was subsequently estimated using factor analysis based on the tetrachoric correlation matrix. Finally, a single latent physiological dysregulation factor was specified in a structural equation model with the six symptom indicators as reflective indicators.

#### Stability of the measurement model across sex

As a sensitivity analysis, we examined the stability of the measurement model across sex using multigroup structural equation models. The sample comprised 599 women and 137 men. Multigroup SEMs with factor loadings and measurement intercepts constrained to equality across sex were estimated. Because the multigroup models did not converge reliably, formal likelihood-ratio-based tests of measurement invariance were not interpreted.

#### Analysis of gastrointestinal complaints

Because gastrointestinal complaints did not emerge as independent predictors in the primary regression models despite showing substantial correlations with muscle problems, additional analyses were performed to determine whether gastrointestinal complaints belonged to the same latent physiological structure. These analyses included:

- tetrachoric correlations,
- logistic regression models incorporating gastrointestinal symptoms,
- exploratory factor analysis,
- structural equation modeling.

The same analytical strategy was subsequently extended to exploratory analyses of urogenital symptoms within neurocognitive-autonomic symptom domains.

#### Stratified training dataset

To assess robustness, all analyses were repeated in a stratified, randomized training dataset derived from the full sample. Stratification was based on sex and disease duration to ensure proportional representation and minimize sampling bias. Results were directionally consistent across datasets.

#### Ethical consideration

The study analysed anonymized questionnaire data collected within the APAV-ME/CFS research program. All procedures complied with applicable ethical and data-protection regulations. Participant confidentiality was maintained throughout the study.

## Results

### Physiological correlates of muscle problems

The primary regression analysis comprised a multivariable logistic regression including all 14 predefined symptom domains, with muscle problems as the outcome. Breathing problems, flu-like symptoms, and temperature-regulation disorders remained significant independent predictors of muscle problems. Cardiovascular problems showed a positive but non-significant association. Neurocognitive symptoms (brain fog, headaches, sensory hypersensitivity), sleep disorders, susceptibility to infection, gastrointestinal complaints, urogenital symptoms, and (food) intolerance-related symptoms were not independently associated with muscle problems (Table 1).

**Table 1:** Multivariable logistic regression predicting muscle problems (non-robust full model, *n* = 736)

Predictor	OR (95% CI)	p-value
Breathing problems	2.49 (1.42–4.38)	0.001
Flu-like symptoms	1.97 (1.14–3.42)	0.015
Temperature-regulation disorders	1.97 (1.11–3.51)	0.021
Sleep disorders	1.71 (0.91–3.21)	0.096
Sensory hypersensitivity	1.65 (0.88–3.06)	0.117
Cardiovascular problems	1.51 (0.83–2.74)	0.173
Brain fog	1.44 (0.70–2.94)	0.319
Visual disturbances	1.43 (0.82–2.50)	0.208
Temperature intolerance	1.30 (0.73–2.31)	0.380
Susceptibility to infection	1.17 (0.67–2.03)	0.576
Gastrointestinal complaints	1.07 (0.59–1.95)	0.814
(Food) Intolerances	0.88 (0.49–1.58)	0.664
Headaches	0.84 (0.49–1.45)	0.538
Urogenital symptoms	0.74 (0.42–1.32)	0.310

Model fit: LR χ^2^ = 113.01, p < 0.001, Pseudo-R^2^ = 0.201

Abbreviations: *n* = number, OR = Odds Ratio; 95%; CI = 95% Confidence Interval; *p*-value = probability; LR χ^2^ = Likelihood-Ratio-Chi-Quadrat-Test; R2 = statistical measure of how closely the data fit the fitted regression line

To further examine the physiologically motivated symptom cluster, a secondary reduced model was estimated including breathing problems, flu-like symptoms, temperature-regulation disorders, cardiovascular problems, and visual disturbances. These domains were selected based on the physiological symptom architecture underlying the analysis rather than solely on statistical significance in the full model. Four of the five physiological symptom domains were independently associated with muscle problems in the reduced model. Visual disturbances showed a positive but non-significant association. Breathing problems had the strongest association, followed by flu-like symptoms, temperature-regulation disorders, and cardiovascular problems (Table 2).

**Table 2:** Reduced physiological model predicting muscle problems* (*n* = 736; total sample)

Predictor	OR (95% CI)	p-value
Breathing problems	2.92 (1.71–5.00)	<0.001
Flu-like symptoms	2.45 (1.49–4.02)	<0.001
Temperature-regulation disorders	2.35 (1.40–3.95)	0.001
Cardiovascular problems	1.87 (1.09–3.24)	0.024
Visual disturbances	1.59 (0.95–2.66)	0.076

Model fit: LR χ^2^ = 102.61, p < 0.001, Pseudo-R^2^ = 0.1825

* The reduced physiological model was specified as a secondary analysis based on the physiological rationale underlying the symptom architecture. It was not a prespecified confirmatory model and the included domains were not selected solely according to statistical significance in the full model

Abbreviations: *n* = number; OR = Odds Ratio; 95% CI = 95% Confidence Interval; *p*-value = probability; LR χ2 = Likelihood-Ratio-Chi-Quadrat-Test; R2 = statistical measure of how closely the data fit the fitted regression line

### Correlation structure of the physiological symptom cluster

Pearson correlations between muscle problems and the five core physiological symptoms ranged from 0.20 to 0.28. Tetrachoric correlations were consistently stronger, indicating substantial associations at the latent level. Muscle problems correlated most strongly with breathing problems (ρ = 0.519), temperature-regulation disorders (ρ = 0.490), and cardiovascular problems (ρ = 0.486) followed by flu-like symptoms (ρ = 0.388) and visual disturbances (ρ = 0.386) (Table 3).

**Table 3:** Tetrachoric correlations with muscle problems (total sample: *n* = 736)

Symptom	Tetrachoric correlation
Breathing problems	0.519
Temperature-regulation disorders	0.490
Cardiovascular problems	0.486
Flu-like symptoms	0.388
Visual disturbances	0.386

### Latent physiological symptom structure

Because the symptom variables were binary, exploratory factor analysis was based on the tetrachoric correlation matrix. All six physiological symptom domains were positively correlated, with pairwise correlations ranging from 0.18 between flu-like and breathing symptoms to 0.60 between breathing and cardiovascular symptoms. The correlation structure was compatible with a predominantly unidimensional representation. In the principal-factor analysis of the tetrachoric correlation matrix, the first eigenvalue was 2.23, accounting for 37.1% of the variance, whereas the second eigenvalue was 0.97 and the remaining eigenvalues ranged from 0.59 to 0.79. Horn’s parallel analysis supported retention of a single component, as only the first adjusted eigenvalue exceeded 1 (adjusted eigenvalue = 2.11; 100 iterations; 95th-percentile criterion).

All six symptom domains loaded positively on the retained factor. Factor loadings ranged from 0.399 for flu-like symptoms to 0.699 for breathing problems; muscle problems loaded at 0.610, cardiovascular problems at 0.677, temperature-regulation disorders at 0.636, and visual disturbances at 0.587. These findings supported a common physiological symptom dimension encompassing muscle, respiratory, cardiovascular, thermoregulatory, visual, and flu-like manifestations.

A complementary one-factor SEM was then estimated. The model showed good approximate fit (RMSEA = 0.046, 90% CI 0.023–0.070; CFI = 0.971; TLI = 0.952; SRMR = 0.026). All six symptom indicators showed significant positive loadings on the latent physiological dysregulation factor (all *p* < 0.001). The loading of muscle problems was fixed at 1.00 for model identification. The unstandardized loadings were 0.737 for flu-like symptoms, 1.832 for breathing problems, 1.393 for cardiovascular problems, 1.303 for temperature-regulation disorders, and 1.427 for visual disturbances (Table 4).

**Table 4:** Exploratory factor analysis and structural equation model loadings of the latent symptom-dysregulation factor in the total sample (*n* = 736)

**Table 4A.** Unstandardized SEM loadings and model fit			
Symptom indicator	Loading	SE	p-value
Muscle problems	1.000*	—	—
Flu-like symptoms	0.737	0.130	<0.001
Breathing problems	1.832	0.205	<0.001
Cardiovascular problems	1.393	0.158	<0.001
Temperature-regulation disorders	1.303	0.152	<0.001
Visual disturbances	1.427	0.180	<0.001

* Loading constrained to 1 for model identification

**Table:** 

**Table 4B.** EFA loadings and standardized SEM loadings		
Symptom domain	EFA loading*	Standardized SEM loading**
Muscle problems	0.610	0.478
Flu-like symptoms	0.399	0.276
Breathing problems	0.699	0.621
Cardiovascular problems	0.677	0.577
Temperature-regulation disorders	0.636	0.515
Visual disturbances	0.587	0.473

Notes: *Principal-factor analysis based on the tetrachoric correlation matrix of the six binary symptom variables. Horn’s parallel analysis (100 iterations; 95th-percentile criterion)supported retention of one factor (observed eigenvalue = 2.228; adjustedeigenvalue = 2.11)

**Standardized loadings calculated from the maximum-likelihood SEM estimates. The loading of muscle problems was fixed to 1.00 for model identification. SEM model fit: χ^2^(9) = 22.91, *p* = 0.006; RMSEA = 0.046 (90% CI0.023–0.070); CFI = 0.971; TLI = 0.952; SRMR = 0.026

Abbreviations: RMSEA = Root Mean Square Error of Approximation; CI = Confidence Interval; CFI = Comparative Fit Index; TLI = Tucker-Lewis Index; SRMR = Standardized Root Mean Square Residual; SEM = Structural equation modelling; EFA = Exploratory factor analysis

### Sex-specific analyses

Sex-stratified regression models suggested different patterns of associations between physiological symptoms and muscle problems. Among women, all five physiological symptom domains were independently associated with muscle problems. Among men, breathing problems and temperature-regulation disorders were associated with muscle problems, whereas flu-like symptoms, cardiovascular problems, and visual disturbances were not statistically significant (Tables 5A and 5B). Differences in statistical significance between the sex-specific models should not, by themselves, be interpreted as evidence that the corresponding regression coefficients differed between women and men (Table 5C).

**Table 5:** Sex- and menopause-specific predictors of muscle problems and SEM-Fit overview (reduced model); A, B and C: sex-specific analyses; D, E and F: analyses restricted to women

A. Women (n = 599)		
Predictor	OR (95% CI)	p
Flu-like symptoms	2.65 (1.54–4.59)	<0.001
Temperature-regulation disorders	2.23 (1.19–4.19)	0.012
Breathing problems	2.18 (1.18–4.00)	0.012
Cardiovascular problems	2.11 (1.15–3.86)	0.016
Visual disturbances	1.98 (1.11–3.51)	0.020

**Table:** 

B. Men (n = 137)*		
Predictor	OR (95% CI)	p
Breathing problems	32.76 (2.71–396.32)	0.006
Temperature-regulation disorders	4.16 (1.24–13.96)	0.021
Flu-like symptoms	2.05 (0.69–6.10)	0.199
Cardiovascular problems	1.04 (0.32–3.36)	0.951
Visual disturbances	0.57 (0.14–2.24)	0.417

* Sex-specific estimates derived from the interaction model. The 95% CI of 2.71 to 396.32 indicates the extremely wide range of uncertainty regarding breathing problems and, consequently, the very imprecise estimate for men

**Table:** 

C. Formal differences between sexes (Symptom-by-sex interaction terms *)		
Symptom ×Sex	Interactions-OR (95% CI)	p
Breathing problems	15.05 (1.16–195.95)	0.038
Visual disturbances	0.29 (0.06–1.27)	0.100
Cardiovascular problems	0.49 (0.13–1.85)	0.294
Temperature-regulation disorders	1.86 (0.48–7.28)	0.371
Flu-like symptoms	0.77 (0.23–2.62)	0.676

* Joint Wald test across all five symptom-by-sex: χ^2^ (5) = 6.77, *p* = 0.238

**Table:** 

D. Women classified as premenopausal (n = 341) and Women classified as postmenopausal based on an age-defined proxy (n = 258)*					
Predictor	≤50 years OR (95% CI)	p	>50 years OR (95% CI)	p	Interaction p
Breathing problems	1.30 (0.53–3.16)	0.565	3.75 (1.50–9.37)	0.005	0.104
Flu-like symptoms	1.56 (0.62–3.94)	0.349	3.29 (1.55–7.00)	0.002	0.220
Temperature-regulation disorders	2.64 (1.04–6.66)	0.040	1.83 (0.76–4.37)	0.175	0.573
Cardiovascular problems	3.69 (1.46–9.30)	0.006	1.38 (0.60–3.17)	0.453	0.120
Visual disturbances	2.90 (1.24–6.75)	0.014	1.44 (0.60–3.45)	0.417	0.265

*Subgroup-specific odds ratios were estimated from a single logistic regression model including menopausal-status-by-symptom interaction terms. Estimates for women aged ≤ 50 years correspond to the symptom main effects. Estimates for women aged > 50 years were obtained as the sum of the symptom main effect and the respective interaction term. Formal differences between groups were assessed using Wald tests of the interaction terms. The joint Wald test of all five menopausal-status-by-symptom interaction terms was not statistically significant (Wald χ^2^ (5) = 5.96, *p* = 0.310)

**Table:** 

E. SEM-fit overview (women classified as premenopausal and women classified as postmenopausal based on an age-defined proxy)								
Subgroup	n	χ^2^ (9)	p	RMSEA (90% CI)	CFI	TLI	SRMR	CD
Premenopausal	341	14.03	0.121	0.040 (0.000–0.079)	0.975	0.959	0.029	0.669
Postmenopausal	258	25.78	0.002	0.085 (0.048–0.124)	0.902	0.836	0.048	0.684

**Table:** 

F. SEM-loadings (women classified as premenopausal and women classified as postmenopausal based on an age-defined proxy)		
Symptom	Premenopausal (n = 341)	Postmenopausal (n = 258)
Muscle problems	1.00 (fixed)	1.00 (fixed)
Flu-like symptoms	0.75	0.60
Breathing problems	1.61	1.88
Cardiovascular problems	1.50	1.11
Temperature-regulation disorders	1.39	1.32
Visual disturbances	1.43	1.53

Abbreviations: *n* = number; OR = Odds Ratio, CI = Confidence Interval; *p*-value = probability value; RMSEA = Root Mean Square Error of Approximation; CFI = Comparative Fit Index; TLI = Tucker-Lewis Index; SRMR = Standardized Root Mean Square Residual; CD = Coefficient of Determination; χ^2^ = Chi-Square Test

Formal sex comparisons were therefore conducted using symptom-by-sex interaction terms in a combined regression model. In sex-specific estimates derived from this interaction model, all five physiological symptom domains were associated with muscle problems among women. Among men, breathing problems (OR = 32.76, 95% CI 2.71–396.32; *p* = 0.006) and temperature-regulation disorders (OR = 4.16, 95% CI 1.24–13.96; *p* = 0.021) were associated with muscle problems, whereas associations with flu-like symptoms, cardiovascular problems, and visual disturbances were not statistically significant. The individual interaction between sex and breathing problems was nominally significant (interaction OR = 15.05, 95% CI 1.16–195.95; *p* = 0.038), but this estimate was highly imprecise. The joint Wald test across all five symptom-by-sex interaction terms was not statistically significant (χ^2^ (5) = 6.77, *p* = 0.238). Thus, the overall evidence did not support a statistically established pattern of effect modification by sex, and subgroup-specific findings should be interpreted as exploratory.

A multigroup SEM was estimated to explore whether the measurement model could be specified similarly across sex. The model constraining factor loadings and measurement intercepts to equality across sex converged and showed acceptable approximate fit (χ^2^ (28) = 55.41, *p* = 0.002; RMSEA = 0.052; CFI = 0.943; TLI = 0.939; SRMR = 0.053). Because the unconstrained multigroup model did not converge, formal likelihood-ratio-based comparisons with constrained models could not be conducted. Therefore, measurement invariance across sex was not formally established. The fit of the constrained model should be interpreted descriptively only.

### Menopause-specific analyses

Exploratory subgroup-specific logistic regression models showed different patterns of associations between physiological symptoms and muscle problems according to menopausal-status group. Among women classified as premenopausal (*n* = 341), cardiovascular problems (OR = 3.69, 95% CI 1.46–9.30; *p* = 0.006), visual disturbances (OR = 2.90, 95% CI 1.24–6.75; *p* = 0.014), and temperature-regulation disorders (OR = 2.64, 95% CI 1.04–6.66; *p* = 0.040) were significantly associated with muscle problems, whereas flu-like symptoms and breathing problems were not. Among women classified as postmenopausal (*n* = 258), breathing problems (OR = 3.75, 95% CI 1,50–9,37; *p* = 0.005) and flu-like symptoms (OR = 3.29, 95% CI 1,55–7,00; *p* = 0.002) were significantly associated with muscle problems, whereas cardiovascular problems, temperature-regulation disorders, and visual disturbances were not. However, these subgroup-specific differences in statistical significance should not be interpreted as evidence of effect modification. In the formal interaction model among women, the joint test of all five menopausal-status-by-symptom interaction terms was not statistically significant (Wald χ^2^ (5)=5.96, *p* = 0.310). Thus, the data do not provide statistical evidence that menopausal status modifies the associations between the five physiological symptoms and muscle problems.

Because age was available only in predefined age categories, an individual-level exclusion of a specific menopausal transition window was not possible. As a sensitivity analysis, the entire 41–50-year age group was excluded, thereby comparing women aged 21–40 years (*n* = 162) with women aged > 50 years (*n* = 258). In the primary interaction model among women, comparing the premenopausal age-proxy group (21–50 years) with the postmenopausal age-proxy group (>50 years), the joint test of all five menopausal-status-by-symptom interaction terms was not statistically significant (Wald χ^2^ (5) = 5.96, *p* = 0.310). In the sensitivity analysis excluding women aged 41–50 years, the interaction between age group and breathing problems was not statistically significant (interaction OR = 3.39, 95% CI 0.68–16.95; *p* = 0.136). The joint robust Wald test of all five age-group-by-symptom interaction terms was likewise not statistically significant (Wald χ^2^ (5) = 3.47, *p* = 0.627).

As the corresponding interaction tests were not statistically significant, these subgroup-specific differences in statistical significance should therefore not be interpreted as evidence of differences between groups. Separate SEM analyses were consistent with a one-factor latent physiological symptom-dysregulation model in both age-defined menopausal-status proxy groups, although model fit differed between the groups. Model fit was better in the premenopausal (χ^2^ (9) = 14.03, *p* = 0.121; RMSEA = 0.040, 90% CI 0.000–0.079; CFI = 0.976; TLI = 0.959; SRMR = 0.029), than in the postmenopausal group (χ^2^ (9) = 25.78, *p* = 0.002; RMSEA = 0.085, 90% CI 0.048–0.124; CFI = 0.902; TLI = 0.836; SRMR = 0.048) (Table 5D). So, the one-factor representation showed good fit among women in the premenopausal age-proxy group but more limited fit among women in the postmenopausal age-proxy group and poor fit among men. Therefore, the findings do not establish measurement invariance across sex or menopausal-status proxy groups. Because formal multigroup measurement-invariance testing across these groups was not performed, the separate subgroup models do not establish that the factor had the same measurement structure in both menopausal-status proxy groups.

Among the individual interaction terms, the interaction between age-defined menopausal status among women and breathing problems yielded the largest interaction estimate, although it did not reach conventional statistical significance (interaction OR = 2.89, 95% CI 0.80–10.36; *p* = 0.104)^1^. Breathing problems showed a stronger SEM loading among postmenopausal than among women in the premenopausal age-proxy groups (1.88 vs. 1.61). Flu-like symptoms, in contrast, showed a somewhat stronger loading among the women classified as premenopausal (0.75 vs. 0.60), a pattern also observed in the corresponding factor analyses. Cardiovascular symptoms showed a stronger SEM loading among women in the premenopausal age-proxy group (1.50 vs. 1.11), whereas visual disturbances had a somewhat stronger loading among women classified as postmenopausal based on an age-defined proxy (1.53 vs. 1.45). Loadings for temperature-regulation disorders were similar between the groups (1.39 vs. 1.32). So, the unstandardized loadings differed descriptively between the separately estimated subgroup models. Formal comparisons of these loadings were not conducted. Therefore, these differences should not be interpreted as statistically established group differences.

Notably, the divergence between SEM loadings and regression estimates for flu-like symptoms is informative. In women classified as premenopausal, flu-like symptoms showed a clear loading on the latent physiological dysregulation factor (SEM loading = 0.75) but were not independently associated with muscle problems in multivariable regression (OR = 1.56, 95% CI 0.62–3.94, *p* = 0.349), whereas temperature-regulation disorders, cardiovascular problems, and visual disturbances showed statistically significant associations with muscle problems. In women classified as postmenopausal based on an age-defined proxy, flu-like symptoms showed a somewhat lower SEM loading (0.60) but were significantly associated with muscle problems (OR = 3.29, 95% CI 1.55–7.00, *p* = 0.002), while cardiovascular, visual, and temperature-regulation symptoms were not statistically significant in the subgroup-specific regression model. These subgroup-specific regression results suggest different patterns of statistical association within the two age-defined groups. However, the formal joint test of the five menopausal-status-by-symptom interaction terms was not statistically significant (Wald χ^2^ (5) = 5.96, *p* = 0.310), and therefore does not provide statistical evidence that the strength of these associations differed according to age-defined menopausal status (Table 5 E, F).

### Sensitivity analysis excluding women aged 41–50 years

Women aged 41–50 years were excluded to reduce potential misclassification around the menopausal transition. In the restricted sample (*n* = 420), flu-like symptoms (OR 3.61, 95% CI 1.91–6.81; *p* < 0.001) and breathing problems (OR 2.64, 95% CI 1.26–5.51; *p* = 0.010) remained independently associated with muscle problems. In a model including symptom-by-age-group interaction terms, none of the five individual interactions was statistically significant. The joint Wald test of all five interaction terms was also non-significant (χ^2^ (5)=3.47, *p* = 0.627), providing no statistical evidence that the associations between symptoms and muscle problems differed between women aged ≤ 40 years and those aged > 50 years (Table S2).

### Influence of disease duration

Exploratory analyses showed broadly similar patterns of associations across disease-duration groups. The six-indicator SEM showed good fit in the ≤5-year and >10-year groups, but poorer fit in the 6–10-year group, indicating some heterogeneity in model fit across duration strata (Table S3). To assess the robustness of this finding with respect to the categorization of disease duration, we additionally repeated the analysis using a four-category classification (≤2 years, 3–10 years, 11–20 years, and >20 years). The results supported measurement invariance across disease-duration groups. Although one individual loading showed a significant score test, the joint test of all factor loadings was not significant, providing no evidence of systematic metric non-invariance (Table 6).

**Table 6:** Measurement invariance of the muscle factor across disease-duration groups

Analysis / parameter class	Comparison or test	df	χ^2^	*p ***value**	Interpretation
**Primary analysis: three disease-duration groups**					
Disease-duration groups	≤5 years (*n* = 254); 6–10 years (*n* = 168); >10 years (*n* = 314)	–	–	–	Total *N* = 736
Metric invariance	Configural vs. metric model	10	8.19	0.611	No evidence against equality of factor loadings across disease-duration groups
Scalar invariance	Metric vs. scalar model	10	13.62	0.191	No evidence against equality of item intercepts across disease-duration groups
Factor loadings (*mcoef*)	Joint score test	10	8.65	0.565	Overall metric invariance supported
Intercepts (*mcons*)	Joint score test	10	13.84	0.180	Overall scalar invariance supported
**Sensitivity analysis: four disease-duration groups**					
Disease-duration groups	≤2 years (*n* = 117); 3–10 years (*n* = 305); 11–20 years (*n* = 188); ≥21 years (*n* = 126)	–	–	–	Total *N* = 736
Metric invariance	Configural vs. metric model	15	22.05	0.106	No evidence against equality of factor loadings using the alternative duration categorization
Scalar invariance	Metric vs. scalar model	15	12.85	0.614	No evidence against equality of item intercepts using the alternative duration categorization
Factor loadings (*mcoef*)	Joint score test	15	16.92	0.323	Overall metric invariance supported
Intercepts (*mcons*)	Joint score test	15	13.03	0.600	Overall scalar invariance supported
**Indicator-level sensitivity finding**	Temperature-regulation disturbances: factor loading (*mcoef*)	3	8.66	0.034	Possible indicator-specific deviation from metric invariance; the global test of all loadings remained non-significant

Note: The latent *muscle* factor was specified by muscle problems, flu-like symptoms, respiratory problems, cardiovascular problems, temperature-regulation disturbances, and visual disturbances. Metric invariance refers to equality of factor loadings. Scalar invariance refers to equality of factor loadings and item intercepts. The primary analysis used the prespecified three-category disease-duration variable (≤5 years, 6–10 years, >10 years). The four-category classification (≤2 years, 3–10 years, 11–20 years, ≥21 years) was conducted as a sensitivity analysis. Overall, neither analysis provided statistical evidence against metric or scalar measurement invariance. The isolated result for temperature-regulation disturbances should be interpreted cautiously because it was not reflected in the global test of factor-loading invariance

### Gastrointestinal and urogenital symptoms

Gastrointestinal complaints showed a statistically significant positive unstandardized loading on the latent physiological factor (b = 1.795, 95% CI 1.424–2.167; *p* < 0.001). In exploratory factor analysis, gastrointestinal complaints loaded moderately on the first factor (loading = 0.566). The model showed acceptable approximate fit (RMSEA = 0.046, 90% CI 0.039–0.054; CFI = 0.925; SRMR = 0.038). However, gastrointestinal complaints were not independently associated with muscle problems in the robust multivariable logistic regression model (OR = 1.07, 95% CI 0.61–1.88; *p* = 0.802). Consistently, the association was not statistically significant in the robust multivariable linear probability model (β = 0.072, 95% CI −0.487 to 0.630; *p* = 0.802) (Table 7).

**Table 7:** Gastrointestinal complaints and the physiological dysregulation factor (total sample)

Analysis	Result
Tetrachoric correlation with muscle problems	ρ = 0.379; *p* < 0.001
Multivariable logistic regression (robust)	OR = 1.07 (95% CI = 0.61–1.88), *p* = 0.802
Robust linear probability model	β = 0.072 (95% CI −0.487 to 0.630); *p* = 0.802
EFA loading on Factor 1	0.566
SEM loading on latent factor*	b = 1.795 [95% CI = 1.42–2.17]; *p* < 0.001
RMSEA	0.046 [95% CI = 0.039–0.054]
CFI	0.925
TLI	0.913
SRMR	0.038

Unstandardized loading; the muscle-problems loading was fixed to 1.0 for model identification

Abbreviations: OR = Odds Ratio; CI = Confidence Interval; *p*-value = probability; SEM = Structural Equation Modeling; RMSEA = Root Mean Square Error of Approximation; CFI = Comparative Fit Index

Urogenital symptoms were positively correlated with muscle problems at the bivariate level and loaded significantly on the latent physiological factor, but they were not independently associated with muscle problems in the robust multivariable logistic regression model (OR = 0.74, 95% CI 0.42–1.31; *p* = 0.300).

Taken together, these findings support a more central role of gastrointestinal complaints within the physiological symptom factor. Urogenital symptoms appear to share some variance with the broader multisystem symptom pattern but may be less centrally represented and more heterogeneous.

## Discussion

### Core findings

The present study identifies a robust and coherent physiological symptom cluster in ME/CFS, with muscle problems at its core. Across multiple analytic approaches—including logistic regression, tetrachoric correlations, factor analysis, and structural equation modeling—muscle problems consistently co-occurred with breathing difficulties, cardiovascular symptoms, temperature dysregulation, visual disturbances, flu-like symptoms and, in secondary analyses, with gastrointestinal complaints. The total-sample analyses supported a one-factor representation of the selected physiological symptoms. In the specified invariance analyses, there was no overall statistical evidence against comparable factor loadings and observed-variable intercepts across the cross-sectional disease-duration groups. In contrast, the one-factor model showed less satisfactory fit in men and in women classified as postmenopausal using an age-based proxy. Therefore, structural comparability across sex and menopausal-status proxy groups remains uncertain [9, 12, 17].

### Integration with previous ME/CFS literature

Mitochondrial studies reported alterations in mitochondrial membrane potential, reduced ATP production rates, and impaired oxidative phosphorylation in subsets of ME/CFS patients, although findings have been heterogeneous across cohorts [6, 7]. In parallel, metabolomic analyses have revealed altered acylcarnitine profiles and disruptions in acyl-lipid metabolism, consistent with impaired pyruvate dehydrogenase function and inefficiencies in aerobic energy metabolism and substrate utilization [18–20], with emerging evidence that the magnitude and pattern of these metabolic alterations may differ between male and female patients [10].

Beyond intrinsic metabolic alterations, several studies have highlighted abnormalities in neuromuscular activation in ME/CFS patients. In particular, reduced voluntary activation and altered perception of effort have been reported, suggesting that central neural mechanisms may contribute to exertional intolerance and muscle fatigability—although the available evidence base remains limited [3]. Autonomic and vascular dysfunction—including endothelial dysfunction, reduced nitric-oxide bioavailability, impaired vasodilation alongside excessive sympathetic vasoconstriction, and reduced tissue perfusion—has also been implicated, linking muscle symptoms to broader disturbances in vascular and autonomic regulation [4, 21–25]—pathways that are themselves modulated by sex-hormone signaling and by menopause-related vascular and autonomic changes [26–28].

Together, these findings established a framework in which muscle symptoms in ME/CFS arise from a combination of impaired energy metabolism, altered neuromuscular activation, and dysregulated perfusion [1, 2, 9]. Recent studies presented at the 2026 International ME/CFS Conference in Berlin refine this framework by identifying specific metabolic, endothelial, and immunological pathways—such as alterations in skeletal-muscle substrate metabolism, arginine–NO and polyamine dysregulation, GPCR-targeting autoantibodies, and arachidonic-acid pathway disturbances [29–32]—and by revealing sex- and menopause-dependent differences in the clinical expression of these processes. Such sex- and hormone-related modulations of immune, vascular, and autonomic regulation [10, 33] are increasingly recognized as a relevant dimension of ME/CFS pathophysiology and are examined in detail in later sections of this discussion [14].

### Candidate integrative or connecting symptoms

Several symptoms in our dataset appear to function as candidate integrative or connecting symptoms, linking multiple symptom domains and reflecting the downstream expression of interacting physiological processes rather than isolated organ-specific dysfunction. Muscle problems were one of the indicators of the latent physiological dysregulation factor and showed consistent associations with several physiological symptom domains. Although they load most strongly on the muscle factor, they also show substantial associations with autonomic, breathing-related, cardiovascular, and flu-like symptoms. This pattern suggests that muscle symptoms integrate neuro-metabolic, vascular, autonomic, and immunological mechanisms. Such an interpretation is compatible with recent evidence for impaired microcirculatory substrate delivery [29], altered oxygen extraction [25], reduced NO-dependent vasodilation [30], GPCR-targeting autoantibodies affecting autonomic signalling [31, 34], and dysregulated arachidonic-acid metabolism [32]. Together with known abnormalities in neuromuscular activation and autonomic regulation of muscle perfusion [1, 24], these findings position muscle symptoms as a prototypical neuro-metabolic-vascular candidate integrative or connecting symptom in ME/CFS.

Additional such candidate integrative or connecting symptoms may include shortness of breath, temperature dysregulation, cognitive slowing, visual disturbances, and generalized fatigue. These symptoms span multiple physiological domains and are compatible with shared mechanisms involving autonomic regulation [28], microcirculatory function, inflammatory signalling, and metabolic stress responses. This interconnected symptom architecture supports the interpretation that the major symptom clusters observed in ME/CFS may arise from partially overlapping biological processes rather than from isolated dysfunction within individual organ systems.

### Alternative interpretation: the physiological dysregulation factor as a manifestation of a chronic PEM-associated “sickness response”

An alternative interpretation, which is compatible with our findings, is to view the identified physiological dysregulation factor through the lens of post-exertional malaise (PEM). PEM is widely regarded as the hallmark symptom of ME/CFS and can be triggered by physical, cognitive, orthostatic, or emotional exertion [1, 4–6]. Regardless of the nature of the trigger, many patients describe a state resembling a systemic illness response characterized by profound fatigue, muscle pain, muscle weakness, chills, flu-like symptoms, and a worsening of multiple symptom domains. This symptom constellation closely resembles the evolutionarily conserved “sickness response” [35], a coordinated physiological program normally activated in response to infection, tissue injury, or other forms of biological threat.

From this perspective, PEM may represent a maladaptive or chronically activated form of such an illness response. Whereas an acute sickness response is typically self-limiting and resolves once the triggering stimulus has been eliminated, ME/CFS may involve a state in which the underlying signaling pathways fail to fully deactivate or become excessively responsive to repeated stressors. The result could be a persistent tendency to activate a physiological program that is normally protective but becomes functionally disabling when chronically engaged.

Within such a framework, muscle-related symptoms occupy a particularly important position. Muscle tissue is among the most metabolically active tissues in the body and is highly sensitive to alterations in energy availability, oxygen delivery, inflammatory signaling, and systemic stress responses. Commonly reported symptoms such as myalgia, weakness, heaviness, and delayed recovery following exertion can therefore be understood as prominent manifestations of a systemic illness response. Importantly, this response would not be restricted to the skeletal muscles of the limbs. Rather, it could affect all muscle systems throughout the body, including respiratory muscles, cardiac muscle, vascular smooth muscle, and other muscle-dependent physiological functions. Such a model provides a biologically coherent explanation for why muscle-related symptoms in our analyses were closely associated with respiratory complaints, cardiovascular symptoms, temperature regulation disturbances, and other physiological symptom domains. Interestingly, gastrointestinal symptoms also appeared to belong to this broader physiological network. Although they did not independently predict muscle problems in regression analyses, they showed substantial correlations with muscle symptoms and loaded strongly on the latent physiological dysregulation factor. This pattern suggests that gastrointestinal manifestations may represent another downstream expression of the same systemic process rather than an independent symptom domain. Such an interpretation is compatible with the concept of PEM as a body-wide illness response affecting multiple organ systems simultaneously.

From this viewpoint, muscle symptoms may represent the most visible clinical expression of a broader systemic response. Their potential role as an integrative or connecting symptom within the broader symptom architecture is suggested by their consistent associations with symptoms spanning several predefined symptom domains and would arise from their position at the intersection of multiple physiological systems that react to the same underlying danger and stress signals. The latent physiological dysregulation factor identified in our analyses may therefore reflect the symptomatic manifestation of a chronic PEM-associated illness response involving several organ systems simultaneously.

Importantly, this framework does not replace autonomic, vascular, immunological, or metabolic mechanisms. Rather, it provides an overarching conceptual model within which these processes may interact. The particularly strong associations observed in our data between muscle symptoms, respiratory complaints, cardiovascular manifestations, and temperature regulation disturbances continue to support a substantial contribution of autonomic and vascular regulatory pathways. The present interpretation therefore views these mechanisms not as competing explanations but as potential biological mediators of a maladaptive systemic illness response. In this sense, the various symptom clusters observed in ME/CFS may represent different manifestations of a common stress-responsive pathophysiological network rather than entirely independent disease processes.

### Sex- and menopause-specific symptom architecture

One of the findings of this work is the observation of different subgroup-specific patterns of association among women classified as premenopausal and postmenopausal. Women in the premenopausal age-proxy group showed significant associations primarily with cardiovascular, visual, and temperature-regulation symptoms, whereas women classified as postmenopausal based on an age-defined proxy showed significant associations primarily with breathing and flu-like symptoms. However, the formal test of menopausal-status-by-symptom interactions did not provide evidence for statistically significant effect modification. These subgroup-specific patterns should be interpreted cautiously. The analyses were performed separately within each subgroup, and differences in statistical significance between groups do not by themselves establish differences in the corresponding regression coefficients.

The postmenopausal symptom architecture appeared to show partial convergence toward the pattern observed in men, particularly with regard to the prominence of breathing-related symptoms. However, women classified as postmenopausal based on an age-defined proxy continued to show a significant association between muscle problems and flu-like symptoms, whereas this association was not statistically significant in men. This persistence of the flu-like symptom association in women classified as postmenopausal based on an age-defined proxy may indicate that symptom domains related to immune or post-exertional processes remain relevant in this subgroup. However, this interpretation is exploratory and cannot be taken as evidence of a statistically established sex difference.

Estrogen is known to affect immune responsiveness, autonomic regulation, vascular function, and thermoregulation [26–28, 36]. The subgroup-specific patterns observed in our study are compatible with the possibility that hormonal or age-related factors contribute to differences in symptom expression. However, because the formal menopausal-status-by-symptom interaction was not statistically significant, these findings should be regarded as exploratory rather than as evidence of a menopause-related modification of symptom associations.

An additional finding of this study is that women classified as postmenopausal based on an age-defined proxy showed several features [33, 37, 38] that resemble the male phenotype [23–25, 29]. Both groups demonstrated strong associations between muscle problems and breathing-related symptoms, suggesting a greater prominence of respiratory and possibly autonomic-perfusion-related manifestations within the shared physiological dysregulation factor. However, the continued association between muscle problems and flu-like symptoms in women classified as postmenopausal based on an age-defined proxy suggests that convergence toward the male phenotype is only partial.

Recent experimental evidence from passive-transfer models further supports the existence of sex-specific disease mechanisms in ME/CFS and related post-infectious syndromes. Patient-derived IgG has been shown to induce sensory and autonomic dysfunction in experimental systems, suggesting that pathogenic autoantibodies may contribute directly to neuroimmune dysregulation [39]. Emerging evidence further indicates that autoantibody repertoires and their neurobiological effects may differ between women and men and may vary across the female lifespan [40]. Together, these findings provide a rationale for investigating whether hormonal or age-related factors may contribute to differences in symptom expression and underlying biological pathways. The present study, however, does not directly assess hormonal status or these biological mechanisms.

An alternative explanation for the persistence of flu-like symptoms in women classified as postmenopausal based on an age-defined proxy is that this symptom category may partially capture the subjective experience of PEM rather than classical infection-like immune activation. Notably, susceptibility to infection—the second component of the immune symptom cluster identified in our previous analyses—was not associated with muscle symptoms in any of the regression models. This selective association of flu-like symptoms, but not infection susceptibility, argues against a simple interpretation in terms of generalized immune vulnerability and is compatible with the possibility that flu-like symptoms partly capture the experiential and physiological manifestations of post-exertional malaise. Additionally, many patients describe PEM as a state characterized by profound chills, internal coldness, shivering sensations, and generalized illness resembling influenza despite the absence of overt infection [41]. Beyond their flu-like quality, PEM episodes are frequently described in terms that point directly to muscle involvement. The IOM report [42] cites patient accounts in which individuals describe their arms, legs, and entire body as feeling heavy and harder to move, and compare themselves to a battery that can never be fully recharged despite extensive rest and a reduction of activities to the bare essentials. Such descriptions illustrate that the subjective experience commonly labelled ‘fatigue’ in ME/CFS is often phenomenologically a muscle experience-one of heaviness, weakness, and an inability to mobilize force or recover after minimal exertion. From this perspective, muscle symptoms are not merely one component of PEM among others but a central sensory and functional dimension of how PEM is experienced. This phenomenological closeness between fatigue, muscle symptoms, and flu-like sensations adds an experiential dimension to the candidate integrative or connecting symptom interpretation discussed above and supports the view that flu-like symptoms in women classified as postmenopausal based on an age-defined proxy partly reflect PEM-associated muscle and autonomic phenomena rather than a discrete immune-activation profile—in line with the broader interpretation of PEM as a chronic, systemically expressed illness response outlined above.

These interpretations are not mutually exclusive. Autonomic, vascular, immunological, and metabolic abnormalities may represent biological mediators through which a chronic PEM-associated illness response becomes clinically expressed.

### Hormonal and biological interpretation

The shift in symptom architecture observed across the age-defined menopausal-status proxy groups is biologically compatible with mechanisms involving vascular and autonomic regulation. Estrogen is an important modulator of endothelial nitric oxide signaling, microvascular tone, and vascular function [26, 27]. Changes in estrogen exposure during the menopausal transition could therefore plausibly influence these physiological systems. However, because hormonal concentrations and detailed menopausal characteristics were not assessed in the present study, these mechanisms cannot be directly linked to the subgroup patterns observed here.

The menopause transition may also represent a particularly important window for neuroimmune dysregulation. During the pre- and perimenopausal years, fluctuating estradiol concentrations can enhance B-cell activation, antibody production, and inflammatory responsiveness, whereas the concomitant decline in progesterone may reduce neuroprotective and immunoregulatory buffering mechanisms. Such hormonal dynamics [43] could facilitate the generation and persistence of pathogenic autoantibodies and amplify neuroimmune signalling pathways [44]. Genetic factors may further contribute to this susceptibility. In women, incomplete X-chromosome inactivation can result in increased expression of immune-related genes [45], including TLR7 [46], thereby promoting heightened immune responsiveness and a greater propensity for autoantibody formation [47, 48]. While our study does not directly assess these mechanisms, they provide a biologically plausible background for the sex- and menopause-specific symptom configurations observed in our data.

Within this framework, hormonal changes associated with menopause may alter the relative prominence of specific symptom domains without abolishing the underlying physiological dysregulation factor. Changes in estrogen exposure during the menopausal transition have been proposed to affect sympathetic activity, endothelial function, and microvascular regulation. These mechanisms provide a biologically plausible hypothesis for future investigation of the breathing-related pattern observed in women classified as postmenopausal based on an age-defined proxy. However, the present data do not permit these mechanisms to be attributed to the observed subgroup pattern.

Indeed, flu-like symptoms emerged as an independent regression predictor of muscle problems only in the subgroup of women classified as postmenopausal (OR = 3.29 [95% CI: 1,55–7.00], *p* = 0.002), whereas in women classified as premenopausal they loaded substantially on the latent physiological factor but did not reach independent significance (OR = 1.56 [95% CI: 0.62–3.94], *p* = 0.349). This illustrates that the pattern of independent associations differed descriptively between the two age-defined proxy groups. One possible explanation is that menopause does not simply produce a male-like phenotype but rather a distinct configuration in which immune-related and autonomic-vascular features coexist and partly redistribute their statistical signature. Given the non-significant interaction test, this difference should be regarded as exploratory rather than as evidence that menopausal status modifies the associations.

A further consideration concerns the fit of separately estimated unidimensional latent physiological symptom-dysregulation models within the sex and age-defined menopausal-status proxy groups. The fit indices suggest that the one-factor representation described the covariance structure more adequately in women classified as premenopausal based on the age-defined proxy than in women classified as postmenopausal based on the proxy or in men. The one-factor model showed good fit among women classified as premenopausal (RMSEA = 0.040; CFI = 0.975; TLI = 0.959). Fit was more limited among women classified as postmenopausal using an age-based proxy (RMSEA = 0.085; CFI = 0.902; TLI = 0.836) and was poor among men (RMSEA = 0.087; CFI = 0.800; TLI = 0.767). Thus, the covariance structure of the included symptoms was most consistent with a unidimensional representation in the premenopausal group. The poorer fit in the postmenopausal-proxy group and in men suggests that the one-factor model may not capture symptom covariation equally well in these groups. Further analyses, including tests of measurement invariance and alternative factor structures, would be needed to determine whether this reflects additional latent dimensions or other sources of model misfit.

The prominent emergence of flu-like symptoms as an independent predictor in women classified as postmenopausal may reflect continued or even enhanced involvement of immune-related pathways, altered stress-response mechanisms, or aspects of PEM that are experienced as a flu-like illness state (see Sect. “Core findings”). At the same time, the stronger independent contributions of cardiovascular, visual, and thermoregulatory symptoms observed in women classified as premenopausal are compatible with the possibility that hormonal or age-related factors may contribute to differences in which manifestations of the shared physiological dysregulation factor are most clinically prominent at a given life stage.

Collectively, these observations are compatible with the hypothesis that hormonal status may contribute to differences in the expression of a common underlying physiological dysregulation process. This interpretation is further supported by the persistence of a comparable latent factor structure across menopausal groups and by the continued inclusion of gastrointestinal symptoms within the same physiological network despite substantial shifts in the relative prominence of individual symptom domains. However, the absence of a statistically significant overall menopausal-by-symptom interaction means that this interpretation remains exploratory.

An interesting clinical observation that may be compatible with this framework concerns restless legs syndrome (RLS). Epidemiological studies consistently report a substantially higher prevalence of RLS in women than in men, although the biological basis of this sex difference remains incompletely understood [49, 50]. It is conceivable that, in a subset of patients, RLS may share symptom covariance with the latent symptom-dysregulation factor and may also involve autonomic, vascular, and metabolic processes [51]. Sex-specific differences in adipose tissue distribution [52], adrenergic receptor expression, and microvascular regulation may contribute both to female predominance of RLS and to its overlap with disorders characterized by impaired autonomic and vascular regulation [53]. This hypothesis is not addressed directly by the present data and requires mechanistic investigation.

### Biological evidence relevant to the latent symptom-dysregulation factor

Recent microdialysis data presented at the 2026 International ME/CFS Conference in Berlin [29] provide preliminary physiological support for the symptom patterns observed in our postmenopausal and male subgroups. Despite normal systemic insulin sensitivity and normal systemic glucose and lipid responses, ME/CFS patients showed markedly reduced glucose availability in skeletal muscle during oral glucose challenge. This pattern suggests impaired microcirculatory substrate delivery rather than exclusively intrinsic metabolic dysfunction. The lower lactate/pyruvate ratio further suggests preserved or enhanced oxidative glucose utilization, arguing against a dominant defect in glycolytic pathways.

These patterns were broadly consistent with the symptom-based analyses. Among women classified as postmenopausal using an age-based proxy, muscle problems were most strongly associated with breathing difficulties and flu-like symptoms. Among men, the strongest associations were observed for breathing difficulties and temperature-regulation symptoms. Such a constellation may reflect the combined effects of impaired perfusion, autonomic dysregulation, and broader stress-response mechanisms, all of which are compatible with the physiological dysregulation factor identified in our study. The microdialysis findings therefore provide a biologically plausible hypothesis that may help contextualize this symptom architecture and warrants direct investigation in longitudinal biomarker studies.

Independent support for biologically distinct ME/CFS subtypes comes from recent GPCR autoantibody profiling in a large post-infectious cohort presented by Schmitz et al. at the 2026 International ME/CFS Conference in Berlin. Hierarchical clustering of autoantibodies against vasoregulatory, autonomic, and neuro-immunological receptors identified four patient subgroups with distinct biological signatures. These clusters differed systematically in fatigue severity, muscle strength, orthostatic symptoms, endothelial dysfunction (RHI ≤ 1.8), and autonomic symptom burden. Autoantibodies targeting β-adrenergic, α-adrenergic, angiotensin II, endothelin, and muscarinic receptors have been associated with symptom severity and autonomic dysfunction in ME/CFS, with receptor-specific patterns differing between patient subgroups [54]. Earlier studies and reviews identified dysregulation of β-adrenergic and muscarinic receptor autoantibodies and proposed their involvement in autonomic and vascular dysfunction [4, 55], while Cabral-Marques et al. [56] demonstrated broader GPCR autoantibody signatures associated with immune homeostasis. Autoantibodies targeting β-adrenergic, α-adrenergic, angiotensin, endothelin, muscarinic, and nicotinic receptors showed differential associations with symptom severity, indicating subgroup-specific dysregulation of autonomic and vascular control mechanisms [4, 55, 56]. These findings align with our observation of distinct physiological dysregulation patterns and support the hypothesis of the presence of distinct ME/CFS subtypes.

Additional support for a shared physiological pathway in ME/CFS comes from recent multi-omics data presented by Dros et al. at the 2026 International ME/CFS Conference in Berlin [32]. In adolescents with post-acute infectious syndromes (ME/CFS, QFS, and Post-COVID), untargeted metabolomics revealed consistent reductions in LTB4-like arachidonic-acid metabolites, including several DiHETEs and leukotriene derivatives. These metabolites are important regulators of microvascular tone, endothelial function, and inflammatory signalling. Their downregulation across PAIS suggests a common disturbance in arachidonic-acid-dependent vascular and immunometabolic pathways. This observation is compatible with our finding that breathing-related and thermoregulatory manifestations become relatively more prominent in women classified as postmenopausal based on an age-defined proxy and men.

Collectively, these independent studies converge on a pathophysiological framework characterized by impaired microcirculation, autonomic dysregulation, altered endothelial adaptability, and disturbed immunometabolic signalling, broadly consistent with the physiological dysregulation factor identified in our study (Table 8).

**Table 8:** Summary of the studies presented at the 2026 International ME/CFS Conference in Berlin on the biological context of the identified latent factor of symptom dysregulation and its consistency with our model

Study	Biological Axis	Biological Context	Alignment With Our Model
Jauert et al. (2026) [29]	Skeletal muscle microdialysis	Reduced intramuscular glucose availability despite normal systemic insulin sensitivity; preserved lipolysis; normal or lower lactate/pyruvate ratio → microcirculatory substrate and oxygen delivery deficit	Provides a biologically plausible hypothesis relevant to muscle symptoms, breathing problems, and temperature dysregulation; may be relevant to the latent symptom-dysregulation factor
Pipper-Krampl (2026) [30]	Arginine–NO and polyamine axis	Male-specific reductions in arginine, homo-arginine, citrulline and spermidine → reduced NO-dependent vasodilation, impaired endothelial compensation	Provides a biologically plausible pathway that may be relevant to perfusion-related symptom manifestations
Schmitz et al. (2026) [31]	GPCR-targeting autoantibodies	Four AAB-defined subgroups with distinct autonomic and vasoregulatory receptor profiles; correlations with fatigue, muscle strength, orthostatic symptoms, endothelial dysfunction	Independent evidence for autonomic-vascular subtypes and symptom-linked receptor dysregulation
Dros et al. (2026) [32]	Arachidonic-acid metabolism	Consistent reductions in LTB4-like metabolites and DiHETEs across PAIS; dysregulated lipoxygenase and CYP-450 pathways → impaired vascular and immunometabolic signaling	Provides evidence for altered vascular and immunometabolic signalling that may be relevant to the symptom-dysregulation framework

Taken together, these findings can be discussed in relation to the proposed framework of multisystemic neuro-immune dysregulation (MuNID), in which immune activation, autoantibody-mediated neuronal interference, autonomic dysfunction, vascular dysregulation, and altered energy metabolism have been hypothesized to interact [15, 57]. In the present study, however, this framework remains a hypothesis for future investigation rather than a mechanism tested by the symptom-based analyses.

Within this model, sex and hormonal status may influence the clinical expression of this network rather than defining entirely distinct disease mechanisms. Consistent with our findings, women classified as premenopausal exhibited stronger associations with cardiovascular symptoms, visual disturbances, and temperature-regulation disorders, whereas men and women classified as postmenopausal based on an age-defined proxy showed greater prominence of breathing-related symptoms. The persistence of flu-like symptoms in women classified as postmenopausal is compatible with the hypothesis that hormonal or age-related factors may contribute to differences in the relative prominence of specific symptom domains while preserving the underlying physiological dysregulation factor. The convergence of symptom-based analyses with recent immunological, metabolomic [19], and vascular studies provides a broader biological context in which the observed symptom-dysregulation factor may be interpreted. However, the present data do not establish that this latent factor represents a neuroimmune, vascular, metabolic, or other specific pathophysiological mechanism.

### Gastrointestinal and urogenital symptoms

Beyond the core physiological cluster, we examined whether gastrointestinal and urogenital symptoms should be considered part of the same latent structure. Gastrointestinal complaints showed a moderate tetrachoric correlation with muscle problems (ρ = 0.379, *p* < 0.001), which was similar in magnitude to the correlation observed for flu-like symptoms (ρ = 0.388, *p* < 0.001). Gastrointestinal complaints also loaded moderately on the first factor in exploratory factor analysis (loading = 0.566). In the SEM, gastrointestinal complaints showed a statistically significant positive unstandardized loading on the latent physiological dysregulation factor (b = 1.795, 95% CI 1.424–2.167; *p* < 0.001). Although gastrointestinal complaints were not independently associated with muscle problems in multivariable logistic regression (OR = 1.07, 95% CI 0.61–1.88; *p* = 0.802), this attenuation may reflect overlap with other correlated symptoms. Taken together, these findings support the inclusion of gastrointestinal complaints as a component of the broader physiological symptom pattern.

Urogenital symptoms showed a small but statistically significant tetrachoric correlation with muscle problems (ρ = 0.213, *p* = 0.005) but were not independently associated with muscle problems in the robust multivariable logistic regression model (OR = 0.74, 95% CI 0.42–1.31; *p* = 0.300). They were also moderately correlated with several cognitive, sensory, sleep-related, and temperature-regulation symptoms. In the exploratory factor analysis, urogenital symptoms showed a modest loading on the general physiological factor (loading = 0.428) alongside substantial item-specific variance (0.752), suggesting that they share some variance with the broader physiological symptom structure but are less centrally represented than gastrointestinal complaints. Consistently, they loaded significantly on the latent factor in the one-factor SEM (unstandardized b = 1.465, 95% CI 1.111–1.820; *p* < 0.001), although this unstandardized estimate should not be compared directly with other loadings without standardization.

In additional exploratory analyses, urogenital complaints were independently associated with brain fog, sleep disturbances, sensory hypersensitivities, and temperature-regulation disturbances. Tetrachoric correlations with these symptoms were moderate, ranging from 0.39 to 0.45. Exploratory factor analysis and Horn’s parallel analysis supported a one-factor representation of these five symptoms. Urogenital complaints showed a positive but comparatively modest loading on this factor (loading = 0.392) and high unique variance (0.847). This pattern suggests that urogenital complaints share variance with a broader neurocognitive, sensory, sleep-related, and thermoregulatory symptom dimension, while also retaining substantial symptom-specific variance. Because this analysis was exploratory and did not assess biological mechanisms directly, it does not establish a specific neuro-autonomic pathophysiological process.

Therefore, one possible explanation is the clinically heterogeneous set of complaints of the urogenital symptom domain that may arise from different pathophysiological processes. Some symptoms may plausibly be related to pelvic-floor or other urogenital musculoskeletal dysfunction, whereas others may primarily reflect local mucosal, infectious, hormonal, neurological, or bladder-specific mechanisms. This heterogeneity may attenuate the observed association between the aggregated urogenital symptom measure and muscle problems, because only a subset of the included symptoms may share a musculoskeletal component. Accordingly, the comparatively weaker and non-independent association observed in the present analyses should not be interpreted as evidence that urogenital symptoms are unrelated to musculoskeletal processes in all individuals.

Taken together, these findings are compatible with the inclusion of gastrointestinal symptoms, but not urogenital symptoms, in the conceptual model of physiological dysregulation in ME/CFS.

### Disease duration

Across the prespecified three disease-duration groups, there was no statistical evidence against metric invariance (likelihood-ratio test: χ^2^ (10) = 8.19, *p* = 0.611) or scalar invariance (χ^2^ (10) = 13.62, *p* = 0.191). Findings were similar when disease duration was alternatively categorized into four groups (metric invariance: χ^2^ (15) = 22.05, *p* = 0.106; scalar invariance: χ^2^ (15) = 12.85, *p* = 0.614). Although the score test suggested a possible indicator-specific deviation for the loading of temperature-regulation disturbances in the four-group analysis (χ^2^ (3) = 8.66, *p* = 0.034), the joint test of all factor loadings was not significant (χ^2^ (15) = 16.92, *p* = 0.323)

Measurement invariance supports statistical comparability of the specified symptom-factor structure across the duration groups. It does not show that symptoms such as flu-like symptoms, breathing problems, or gastrointestinal complaints necessarily have identical clinical contexts or pathophysiological meanings at different stages of illness (Table 6).

## Strengths and limitations

The study has several strengths, including the use of multiple complementary statistical methods, the consistency of findings across analytic approaches, and the explicit stratification by age-defined menopausal status. The identification of a latent physiological dysregulation factor with excellent model fit in the total sample further strengthens the interpretation that these symptoms share a common latent symptom dimension. Exploratory subgroup analyses stratified by sex, menopausal status, and disease duration enabled a differentiated view of how this shared dysregulation factor is clinically expressed across patient groups.

Several limitations should be acknowledged. The cross-sectional design precludes causal inference, and the dichotomization of symptoms may obscure gradations of severity. Subgroup analyses, particularly in women classified as postmenopausal based on an age-defined proxy and men, were based on smaller sample sizes (*n* = 258 and *n* = 137, respectively) and should therefore be interpreted as exploratory. In addition, several mechanistic findings discussed here derive from conference presentations and await peer-reviewed publication and independent replication. Future work should examine whether these physiological clusters predict functional outcomes, disease trajectories, or treatment responses, and whether they can be replicated in independent cohorts.

The number of secondary and subgroup analyses represents an additional limitation. Although the primary regression analysis was defined a priori around muscle problems as the outcome and included the 14 symptom domains simultaneously, subsequent reduced-model, sex-stratified, age-defined menopausal-status, and disease-duration analyses increased the number of statistical comparisons. No global FDR correction was applied across these heterogeneous analyses. Consequently, findings from secondary and exploratory analyses should be interpreted cautiously, with emphasis on effect sizes and confidence intervals rather than statistical significance alone, and require confirmation in independent cohorts.

A further limitation relates to the operationalization of menopausal status, which was based on an age-based proxy (≤50 vs. >50 years) rather than on direct hormonal measurements or detailed reproductive history. Because age was available only in broad age categories, a more refined exclusion of participants around the menopausal transition could not be performed without making additional assumptions about individual menopausal status. Furthermore, this approach does not capture perimenopausal fluctuations, individual variability in the timing of menopause, or the potential influence of hormone replacement therapy. Consequently, the extent to which the observed subgroup differences reflect hormonal status cannot be determined from the present data. However, the robustness of the main associations after exclusion of women aged 41–50 years further suggests that the observed pattern was not driven solely by women in the potential menopausal transition range. That said, the non-significant interaction tests do not support a statistically established difference in symptom associations between the two age-defined groups. Future studies should employ more refined classifications—incorporating hormone levels, menstrual history, and information on hormone replacement therapy—to clarify the hormonal mediators underlying the observed patterns.

With regard to the structural equation models, fit was excellent for the total sample (RMSEA = 0.046; CFI = 0.971), for the female subgroup, and particularly for women classified as premenopausal (RMSEA = 0.040; CFI = 0.975). However, fit was more limited in women classified as postmenopausal based on an age-defined proxy (RMSEA = 0.085; CFI = 0.902; TLI = 0.836) and poor in men (RMSEA = 0.087; CFI = 0.800; TLI = 0.767). The reduced fit in these subgroups likely reflects smaller sample sizes but may also indicate that the strictly unidimensional latent-factor model captures the symptom structure less precisely in these populations. Future studies with larger postmenopausal and male cohorts should investigate whether multi-factor solutions provide better fit and reveal additional substructure—such as a secondary immuno-inflammatory or PEM-related subdimension in women classified as postmenopausal or a more centralized autonomic-respiratory pole in men.

An additional limitation concerns the interpretation of the identified physiological dysregulation factor. The discussion proposes that this factor may reflect a chronic PEM-associated illness or danger response. However, this interpretation represents a conceptual framework derived from observed symptom associations rather than a directly tested hypothesis. The present dataset does not include longitudinal assessments of PEM episodes or biological measures of immune activation, autonomic function, vascular regulation, or metabolic processes. Consequently, the proposed framework cannot be directly validated using the current data. The findings therefore do not allow conclusions regarding whether the latent factor reflects a single underlying mechanism or the convergence of several interacting pathophysiological processes. Future studies combining longitudinal symptom assessments with physiological and molecular biomarkers will be required to evaluate these hypotheses.

Finally, although the analyses were repeated in a stratified training dataset to assess robustness and yielded directionally consistent results, true external validation in an independent ME/CFS cohort remains essential. Replication in cohorts using different diagnostic criteria, recruitment strategies, or geographic settings would substantially strengthen the generalizability of the identified physiological dysregulation factor and its sex- and menopause-specific expressions.

## Conclusion

In conclusion, this cross-sectional study identified a latent physiological symptom-dysregulation factor linking muscle problems with respiratory, cardiovascular, thermoregulatory, visual, and flu-like symptoms in individuals with ME/CFS. Secondary analyses further supported the inclusion of gastrointestinal complaints in the broader physiological symptom pattern. Across multiple analytical approaches, muscle symptoms emerged as a central candidate integrative or connecting symptom within the broader symptom architecture connecting several physiological domains, supporting the view that they reflect a broader multisystem process rather than an isolated disorder of skeletal muscle.

The observed symptom patterns differed descriptively between women classified as premenopausal and postmenopausal according to an age-based proxy. Women classified as premenopausal showed significant associations primarily with cardiovascular, visual, and thermoregulatory symptoms, whereas women classified as postmenopausal based on an age-defined proxy showed significant associations primarily with breathing and flu-like symptoms. However, the formal test of the five menopausal-status-by-symptom interactions was not statistically significant. These subgroup-specific patterns should therefore be regarded as exploratory rather than as evidence of a statistically demonstrated menopause-related modification of symptom associations.

Overall, the results were consistent with statistical comparability of the specified linear symptom-factor model across the cross-sectional disease-duration groups examined. The one-factor model showed less satisfactory fit in men and in women classified as postmenopausal using an age-defined proxy. Therefore, structural comparability across sex and menopausal-status proxy groups remains uncertain. Although the relative prominence of individual symptoms differed descriptively across sex and age-defined menopausal-status groups, the formal interaction tests did not provide consistent evidence of effect modification.

This finding does not demonstrate longitudinal stability within individuals or establish that identically labelled symptoms have the same clinical or pathophysiological meaning at different stages of illness. Symptoms with the same label may occur in different clinical contexts or reflect different underlying processes at different stages of disease. The present findings therefore support the statistical comparability of the specified symptom dimension across disease-duration groups, but do not establish invariance of the clinical significance or underlying mechanisms of individual symptoms. This distinction should be considered when interpreting longitudinal changes or treatment effects in future clinical studies.

Taken together, our findings support the inclusion of sex and age-defined menopausal-status proxies in future ME/CFS phenotyping strategies and highlight the importance of investigating physiological dysregulation as an integrated, multisystem phenomenon. Future longitudinal studies integrating symptom trajectories, PEM assessments, physiological measures, and molecular biomarkers will be required to determine whether and how the identified symptom-dysregulation factor relates to specific biological pathways underlying disease heterogeneity in ME/CFS.

## Electronic supplementary material

Below is the link to the electronic supplementary material.

Supplementary Material 1

Supplementary Material 2

Supplementary Material 3

## Data Availability

All data supporting the results of this study are included in the article or can be requested from the corresponding author.

## Footnotes

1 Similar results were obtained after additionally accounting for disease duration group. When disease duration was categorized into three groups, the interaction OR was 2.94 (95% CI 0.87–9.96; *p* = 0.084); with four disease-duration categories, it was 3.13 (95% CI 0.92–10.67; *p* = 0.068).

## Declarations

### Additional notes

The translation from German was supported by http://www.DeepL.com/Translator (free version). Furthermore, we would like to thank the AI tool ChatGPT (OpenAI) for language editing and wording optimization in individual sections under human guidance. All ideas, data and interpretations originate from the authors.

## References

[1] Wirth KJ, Scheibenbogen C. Pathophysiology of skeletal muscle disturbances in myalgic encephalomyelitis/chronic fatigue syndrome (ME/CFS). J Transl Med. 2021;19(1):162. 10.1186/s12967-021-02833-2. PMC8058748 33882940

[2] Scheibenbogen C, Wirth KJ. Key pathophysiological role of skeletal muscle disturbance in post COVID and myalgic encephalomyelitis/chronic fatigue syndrome (ME/CFS): accumulated evidence. J Cachexia, Sarcopenia Muscle. 2025;16(1):e13669. 10.1002/jcsm.13669. PMC11671797 39727052

[3] Nuzzo JL, Taylor JL, Latella C. Muscle strength, muscle endurance, voluntary activation, and perception of effort in myalgic encephalomyelitis/chronic fatigue syndrome (ME/CFS): an overview. Fatigue: Biomed, Health Behav. 2026;14(1):42–60. 10.1080/21641846.2025.2556941.

[4] Wirth K, Scheibenbogen C. A unifying hypothesis of the pathophysiology of myalgic encephalomyelitis/chronic fatigue syndrome (ME/CFS): recognitions from the finding of autoantibodies against ß2-adrenergic receptors. Autoimmun Rev. 2020;19(6):102527. 10.1016/j.autrev.2020.102527. 32247028

[5] Barhorst EE, Boruch AE, Cook DB, Lindheimer JB. Pain-related post-exertional malaise in myalgic encephalomyelitis / chronic fatigue syndrome (ME/CFS) and fibromyalgia: a systematic review and three-level meta-analysis. Pain Med. 2022;23(6):1144–57. 10.1093/pm/pnab308. 34668532

[6] Syed AM, Karius AK, Ma J, Wang PY, Hwang PM. Mitochondrial dysfunction in myalgic encephalomyelitis/chronic fatigue syndrome. Physiol (Bethesda). 2025;40(4):0. 10.1152/physiol.00056.2024. PMC12151296 39960432

[7] Missailidis D, Annesley SJ, Fisher PR. Pathological mechanisms underlying myalgic encephalomyelitis/chronic fatigue syndrome. Diagn (Basel). 2019;9(3):80. 10.3390/diagnostics9030080. PMC6787592 31330791

[8] Renz-Polster H, Tremblay ME, Bienzle D, Fischer JE. The pathobiology of myalgic encephalomyelitis/chronic fatigue syndrome: the case for neuroglial failure. Front Cell Neurosci. 2022;16:888232. 10.3389/fncel.2022.888232. PMC9124899 35614970

[9] Arron HE, Marsh BD, Kell DB, Khan MA, Jaeger BR, Pretorius E. Myalgic encephalomyelitis/chronic fatigue syndrome: the biology of a neglected disease. Front. Immunol. 2024;15:1386607. 10.3389/fimmu.2024.1386607. PMC11180809 38887284

[10] Thomas N, Gurvich C, Huang K, Gooley PR, Armstrong CW. The underlying sex differences in neuroendocrine adaptations relevant to myalgic encephalomyelitis chronic fatigue syndrome. Front Neuroendocrinol. 2022;66:100995. 10.1016/j.yfrne.2022.100995. 35421511

[11] Habermann-Horstmeier L, Horstmeier LM. Symptome der myalgischen enzephalomyelitis/des chronischen fatigue-syndroms (ME/CFS) im Krankheitsverlauf. Präv Gesundheitsf. 2025. 10.1007/s11553-025-01280-x.

[12] Habermann-Horstmeier L, Horstmeier LM. Symptom clusters in ME/CFS reflect distinct neuroimmune and autonomic pathophysiological mechanisms: a translational model. J Transl Med. 2026;24:606. 10.1186/s12967-026-08159-1. PMC13126800 42050709

[13] Habermann-Horstmeier L, Horstmeier LM. Sex-specific differences in neurocognitive-sensory and autonomic ME/CFS symptomatology: evidence from structural equation modeling with implications for a pathophysiological model. Under Rev Bull Natl Res Cent.

[14] Habermann-Horstmeier L, Horstmeier LM. Sex- and menopause-related differences in immune and gastrointestinal symptom architecture in ME/CFS:evidence from factor analysis and structural equation modeling. J Transl Med. 2026;24(1):970 10.1186/s12967-026-08699-6 PMC13418152 42522006

[15] Carruthers BM, Jain AK, De Meirleir KL, et al. Myalgic encephalomyelitis/chronic fatigue syndrome: clinical working case definition, diagnostic and treatment protocols. J Chronic Fatigue Syndr. 2003;11(1):7–115. 10.1300/J092v11n01_02. https://www.ncbi.nlm.nih.gov/books/NBK274235/pdf/Bookshelf_NBK274235.pdf. 5 September 2026.

[16] Carruthers BM, van de Sande MI, De Meirleir KL, et al. Myalgic encephalomyelitis: international consensus criteria. J Intern Med. 2011;270(4):327–38. 10.1111/j.1365-2796.2011.02428.x. PMC3427890 21777306

[17] Heng B, Gunasegaran B, Krishnamurthy S, Bustamante S, Pires AS, Chow S, et al. Mapping the complexity of ME/CFS: Evidence for abnormal energy metabolism, altered immune profile, and vascular dysfunction. Cell Rep Med. 2025;6(12):102514. 10.1016/j.xcrm.2025.102514 (accessed on 5 September 2026). PMC12765951 41406947

[18] Fluge Ø, Mella O, Bruland O, Risa K, Dyrstad SE, Alme K, et al. Metabolic profiling indicates impaired pyruvate dehydrogenase function in myalgic encephalopathy/chronic fatigue syndrome. JCI Insight. 2016;1(21):e89376. 10.1172/jci.insight.89376. PMC5161229 28018972

[19] Germain A, Barupal DK, Levine SM, Hanson MR. Comprehensive circulatory metabolomics in ME/CFS reveals disrupted metabolism of acyl lipids and steroids. Metabolites. 2020;10(1):34. 10.3390/metabo10010034. PMC7023305 31947545

[20] Che X, Brydges CR, Yu Y, Price A, Joshi S, Roy A, et al. Metabolomic evidence for peroxisomal dysfunction in myalgic Encephalomyelitis/Chronic fatigue syndrome. Int J Mol Sci. 2022;23(14):7906. 10.3390/ijms23147906. PMC9320121 35887252

[21] Blauensteiner J, Bertinat R, León LE, Riederer M, Sepúlveda N, Westermeier F. Altered endothelial dysfunction-related miRs in plasma from ME/CFS patients. Sci Rep. 2021;11(1):10604. 10.1038/s41598-021-89834-9. PMC8134566 34011981

[22] Bertinat R, Villalobos-Labra R, Hofmann L, Blauensteiner J, Sepúlveda N, Westermeier F. Decreased NO production in endothelial cells exposed to plasma from ME/CFS patients. Vascul Pharmacol. 2022;143:106953. 10.1016/j.vph.2022.106953. 35074481

[23] Sandvik MK, Sørland K, Leirgul E, Rekeland IG, Stavland CS, et al. Endothelial dysfunction in ME/CFS patients. PLoS One. 2023;18(2):e0280942. 10.1371/journal.pone.0280942. PMC9894436 36730360

[24] Clmc VC, Verheugt FWA, Rowe PC, Visser FC. Cerebral blood flow is reduced in ME/CFS during head-up tilt testing even in the absence of hypotension or tachycardia: a quantitative, controlled study using doppler echography. Clin Neurophysiol Pract. 2020;5:50–58. 10.1016/j.cnp.2020.01.003. PMC7044650 32140630

[25] Joseph P, Arevalo C, Oliveira RKF, Faria-Urbina M, Felsenstein D, Oaklander AL, et al. Insights from invasive cardiopulmonary exercise testing of patients with myalgic encephalomyelitis/chronic fatigue syndrome. Chest. 2021;160(2):642–51. 10.1016/j.chest.2021.01.082. PMC8727854 33577778

[26] Moreau KL, Hildreth KL. Vascular aging across the menopause transition in healthy women. Adv Vasc Med. 2014;2014:204390. 10.1155/2014/204390. PMC4433172 25984561

[27] SenthilKumar G, Katunaric B, Bordas-Murphy H, Sarvaideo J, Freed JK. Estrogen and the vascular endothelium: the unanswered questions. Endocrinology. 2023;164(6):bqad079. 10.1210/endocr/bqad079. PMC10230790 37207450

[28] Lee J, Keller-Ross ML. Menopause and its effects on autonomic regulation of blood pressure: insights and perspectives. Auton Neurosci. 2025;260:103295. 10.1016/j.autneu.2025.103295. PMC12219667 40460599

[29] Jauert N, Schilling-Ziese K, Meyer A, Heigl M, Scheibenbogen C, Doehner W, et al. Skeletal Muscle Metabolism in Women with ME/CFS. Poster presented at: International ME/CFS Conference. Berlin, Germany: 2026 May. https://events.mecfs-research.org/de/events/conference_2026/posters.

[30] Pipper-Krampl C, Jeremic D, Wagner B, Hausl M, Raml R, Eberl A, et al. Sex‑specific alterations in arginine‑NO and polyamine metabolism in ME/CFS. Poster presented at: International ME/CFS Conference. Berlin: 2026. https://events.mecfs-research.org/de/events/conference_2026/posters.

[31] Schmitz J, Sotzny F, Kedor C, et al. Distinct autoantibody profiles targeting G‑protein‑coupled receptors in subgroups of a large post‑infectious ME/CFS cohort. Poster presented at: International ME/CFS Conference. Berlin, Germany: 2026 May. https://events.mecfs-research.org/de/events/conference_2026/posters.

[32] Dros M, Mocking TR, Vroegindeweij A, et al. Dysregulated arachidonic acid metabolism is a shared feature among post‑acute infectious syndromes: preliminary results from the QVS study. Poster presented at: International ME/CFS Conference. Berlin, Germany: 2026 May. https://events.mecfs-research.org/de/events/conference_2026/posters.

[33] Klein SL, Flanagan KL. Sex differences in immune responses. Nat Rev Immunol. 2016;16(10):626–38. 10.1038/nri.2016.90. 27546235

[34] Cabral-Marques O, Schimke LF, Moll G, et al. Advancing research on regulatory autoantibodies targeting GPCRs: insights from the 5th international symposium. Autoimmun Rev. 2025;24(9):103855. 10.1016/j.autrev.2025.103855. 40543860

[35] Watkins LR, Maier SF. Implications of immune-to-brain communication for sickness and pain. Proc Natl Acad Sci U S A. 1999;96(14):7710–13. 10.1073/pnas.96.14.7710. PMC33606 10393885

[36] Thurston RC. Vasomotor symptoms: natural history, physiology, and links with cardiovascular health. Climacteric. 2018;21(2):96–100. 10.1080/13697137.2018.1430131. PMC5902802 29390899

[37] Gubbels Bupp MR. Sex, the aging immune system, and chronic disease. Cell Immunol. 2015;294(2):102–10. 10.1016/j.cellimm.2015.02.002. 25700766

[38] Hoffmann J, Liu J, Seddu K, et al. Sex hormone signaling and regulation of immune function. Immunity. 2023;56(11):2472–91. 10.1016/j.immuni.2023.10.008. 37967530

[39] de Sá K, Silva J, Bayarri-Olmos R, et al. A causal link between autoantibodies and neurological symptoms in long COVID. Cell. 2026;189(11):3214–35.e37. 10.1016/j.cell.2026.04.042. PMC13501478 42208499

[40] Angum F, Khan T, Kaler J, Siddiqui L, Hussain A. The prevalence of autoimmune disorders in women: a narrative review. Cureus. 2020;12(5):e8094. 10.7759/cureus.8094. PMC7292717 32542149

[41] Johns Hopkins Medicine. What is post-exertional malaise? https://www.hopkinsmedicine.org/-/media/johns-hopkins-childrens-center/documents/specialties/adolescent-medicine/cfs-pem-info.pdf. 20 June 2026.

[42] IOM - Committee on the Diagnostic Criteria for Myalgic Encephalomyelitis/Chronic Fatigue Syndrome. Board on the health of select populations. Beyond Myalgic Encephalomyelitis/ Chronic Fatigue Syndrome: Redefining an Illness. Washington (DC): Institute of Medicine: National Academies Press (US); 2015. 10.17226/19012. 25695122

[43] Flanagan KL, Klein SL. Knowledge gaps and research priorities to understand sex differences in immunity. PLoS Biol. 2026;24(2):e3003578. 10.1371/journal.pbio.3003578. PMC12863523 41628075

[44] Bouman A, Heineman MJ, Faas MM. Sex hormones and the immune response in humans. Hum Reprod Update. 2005;11(4):411–23. 10.1093/humupd/dmi008. 15817524

[45] Libert C, Dejager L, Pinheiro I. The X chromosome in immune functions: when a chromosome makes the difference. Nat Rev Immunol. 2010;10:594–604. 10.1038/nri2815. 20651746

[46] Souyris M, Cenac C, Azar P, Daviaud D, Canivet A, Grunenwald S, et al. TLR7 escapes X chromosome inactivation in immune cells. Sci Immunol. 2018;3(19):eaap8855. 10.1126/sciimmunol.aap8855. 29374079

[47] Dou D, Zhao Y, Belk J, et al. Xist ribonucleoproteins promote female sex-biased autoimmunity. Cell. 2024;187(3):733–49.e16. 10.1016/j.cell.2023.12.037. PMC10949934 38306984

[48] Lahita RG. Sex and gender influence on immunity and autoimmunity. Front Immunol. 2023;14:1142723. 10.3389/fimmu.2023.1142723. PMC10250588 37304263

[49] Broström A, Alimoradi Z, Lind J, Ulander M, Lundin F, Pakpour A. Worldwide estimation of restless legs syndrome: a systematic review and meta-analysis of prevalence in the general adult population. J Sleep Res. 2023;32(3):e13783. 10.1111/jsr.13783. 36600470

[50] Seeman MV. Why are women prone to restless legs syndrome? Int J Environ Res Public Health. 2020;17(1):368. 10.3390/ijerph17010368. PMC6981604 31935805

[51] Innes KE, Selfe TK, Agarwal P. Restless legs syndrome and conditions associated with metabolic dysregulation, sympathoadrenal dysfunction, and cardiovascular disease risk: a systematic review. Sleep Med Rev. 2012;16(4):309–39. 10.1016/j.smrv.2011.04.001. 21733722

[52] Lafontan M, Berlan M. Fat cell adrenergic receptors and the control of white and brown fat cell function. J Lipid Res. 1993;34(7):1057–91. 10.1016/S0022-2275(20)37695-1. https://pubmed.ncbi.nlm.nih.gov/8371057/. 8371057

[53] Gossard TR, Trotti LM, Videnovic A, St Louis EK. Restless legs syndrome: contemporary diagnosis and treatment. Neurotherapeutics. 2021;18(1):140–55. 10.1007/s13311-021-01019-4. PMC8116476 33880737

[54] Freitag H, Szklarski M, Lorenz S, Sotzny F, Bauer S, Philippe A, et al. Autoantibodies to vasoregulative G-protein-coupled receptors correlate with symptom severity, autonomic dysfunction and disability in myalgic encephalomyelitis/chronic fatigue syndrome. J Clin Med. 2021;10(16):3675. 10.3390/jcm10163675. PMC8397061 34441971

[55] Bynke A, Julin P, Gottfries CG, Heidecke H, Scheibenbogen C, Bergquist J. Autoantibodies to beta-adrenergic and muscarinic cholinergic receptors in myalgic encephalomyelitis (ME) patients - a validation study in plasma and cerebrospinal fluid from two Swedish cohorts. Brain Behav Immun Health. 2020;7:100107. 10.1016/j.bbih.2020.100107. PMC8474431 34589868

[56] Cabral-Marques O, Marques A, Giil LM, De Vito R, Rademacher J, Günther J, et al. GPCR-specific autoantibody signatures are associated with physiological and pathological immune homeostasis. Nat Commun. 2018;9(1):5224. 10.1038/s41467-018-07598-9. PMC6283882 30523250

[57] NIH (National Institutes of Health). Report of the ME/CFS research roadmap working group of council. 2024, May, 15. https://www.ninds.nih.gov/sites/default/files/2024-05/Report%20of%20the%20MECFS%20Research%20Roadmap%20Working%20Group%20of%20Council_508C.pdf. 20 June 2026.

