# Supplementary material for: Muscle symptoms and a latent physiological symptom-dysregulation factor in ME/CFS: exploratory analyses by sex and age-defined menopausal-status proxy group
Source: J Transl Med. 2026 Sep 28;24:1216. doi: 10.1186/s12967-026-09018-9 (PMC13621514; doi:10.1186/s12967-026-09018-9)
Supplement: Supplementary file 1 [file 12967_2026_9018_MOESM1_ESM.docx]

**Supplementary Table S1**

| **Symptom variable** | **Operational definition** |
| --- | --- |
| Brain fog | Memory problems, word-finding difficulties, or brain fog |
| Headaches | Headaches, e.g. migraine-like headaches |
| Muscle problems | Muscle pain, post-exertional muscle soreness, twitching, or cramps |
| Visual disturbances | Blurred/unclear vision, tunnel vision, zig-zag lines |
| Flu-like symptoms | Flu-like symptoms |
| Susceptibility to infection | Increased susceptibility to infections with prolonged recovery |
| Gastrointestinal complaints* | Bloating, diarrhea, intestinal cramps |
| Urogenital complaints** | Sudden urinary urgency and related urinary/reproductive complaints |
| (Food) intolerances | Intolerances to foods, medications, or chemicals |
| Cardiovascular symptoms | Palpitations, blood-pressure fluctuations, dizziness during prolonged standing or positional changes |
| Breathing problems | Respiratory difficulties, e.g. during stair climbing or prolonged speaking |
| Sleep disturbances | Sleep-maintenance problems or altered day/night rhythm |
| Sensory hypersensitivity | Hypersensitivity to light, sound, odors, or touch |
| Temperature-regulation disturbances | Cold sensation, chills, hot flashes |
| Temperature intolerance | Intolerance of high or low ambient temperature |
|  |  |

| **Predefined Symptom Cluster** | **Definition** |
| --- | --- |
| *Brain* | Brain fog, sensory hypersensitivity, visual disturbances, sleep disturbances, headaches |
| *Autonomic** | Cardiovascular problems, breathing problems, temperature-regulation disturbances, gastrointestinal complaints |
| *Immune* | flu-like symptoms, susceptibility to infections |
| *Gut** | gastrointestinal complaints, (food) intolerances |
|  |  |

* Gastrointestinal complaints were included in both the Autonomic and Gut symptom clusters according to the previously published cluster framework. **Urogenital complaints were not assigned to any of the four predefined clusters
