# Supplementary material for: Muscle symptoms and a latent physiological symptom-dysregulation factor in ME/CFS: exploratory analyses by sex and age-defined menopausal-status proxy group
Source: J Transl Med. 2026 Sep 28;24:1216. doi: 10.1186/s12967-026-09018-9 (PMC13621514; doi:10.1186/s12967-026-09018-9)
Supplement: Supplementary file 2 [file 12967_2026_9018_MOESM2_ESM.docx]

**Supplementary Table S2.** Sensitivity analysis: women aged < 41 years (n = 162) and women aged > 50 years (n = 258) are compared. Women aged 41–50 years were excluded.

| **Predictor** | **<41 years OR (95% CI)** | **p** | **>50 years OR (95% CI)** | **p** | **Interaction p** |
| --- | --- | --- | --- | --- | --- |
| Breathing problems | 1.10 (0.29–4.13) | 0.883 | 3.75 (1.57–8.96) | 0.003 | 0.136 |
| Flu-like symptoms | 4.29 (1.13–16.29) | 0.033 | 3.29 (1.53–7.07) | 0.002 | 0.734 |
| Temperature-regulation disorders | 1.35 (0.30–6.10) | 0.694 | 1.83 (0.76–4.40) | 0.178 | 0.735 |
| Cardiovascular problems | 3.02 (0.72–12.69) | 0.131 | 1.38 (0.52–3.64) | 0.520 | 0.353 |
| Visual disturbances | 1.86 (0.52–6.67) | 0.340 | 1.44 (0.60–3.44) | 0.408 | 0.748 |

* Subgroup-specific odds ratios were estimated in one logistic-regression model containing age-group-by-symptom interaction terms, with robust standard errors. Estimates for women aged <41 years are the symptom main effects. Estimates for women aged >50 years combine the respective symptom main effect and interaction term. Individual interaction p-values are Wald tests of the relevant interaction coefficients. The joint Wald test of all five interaction terms was not statistically significant: χ²(5) = 3.47, p = 0.627.
