# Supplementary material for: Muscle symptoms and a latent physiological symptom-dysregulation factor in ME/CFS: exploratory analyses by sex and age-defined menopausal-status proxy group
Source: J Transl Med. 2026 Sep 28;24:1216. doi: 10.1186/s12967-026-09018-9 (PMC13621514; doi:10.1186/s12967-026-09018-9)
Supplement: Supplementary file 3 [file 12967_2026_9018_MOESM3_ESM.docx]

| . use "C:\Users\Lotte\Documents\Symptome - Krankheitsdauer, Krankheitsgruppen\Zusammengefhrte Daten\training.dta", clear |  |  |
| --- | --- | --- |
| . logit Muskelprobleme GrippehnlicheSymptome Atemprobleme HerzKreislaufProbleme Temperregulierungsstrungen Sehstrungen |  |  |
| Iteration 0: log likelihood = -215.03446 |  |  |
| Iteration 1: log likelihood = -179.84265 |  |  |
| Iteration 2: log likelihood = -173.30795 |  |  |
| Iteration 3: log likelihood = -173.24504 |  |  |
| Iteration 4: log likelihood = -173.24498 |  |  |
| Iteration 5: log likelihood = -173.24498 |  |  |
| Logistic regression Number of obs = 512 |  |  |
| LR chi2(5) = 83.58 |  |  |
| Prob > chi2 = 0.0000 |  |  |
| Log likelihood = -173.24498 Pseudo R2 = 0.1943 |  |  |
|  |  |  |
| Muskelprobleme Coef. Std. Err. z P>z [95% Conf. Interval] |  |  |
| GrippehnlicheSymptome .9919999 .2898862 3.42 0.001 .4238333 1.560166 |  |  |
| Atemprobleme 1.245409 .322207 3.87 0.000 .6138947 1.876923 |  |  |
| HerzKreislaufProbleme .5423693 .3165021 1.71 0.087 -.0779635 1.162702 |  |  |
| Temperregulierungsstrungen .8236596 .3034337 2.71 0.007 .2289405 1.418379 |  |  |
| Sehstrungen .2510061 .3041925 0.83 0.409 -.3452003 .8472124 |  |  |
| _cons -.6233797 .3009746 -2.07 0.038 -1.213279 -.0334803 |  |  |
|  |  |  |
| . pwcorr Muskelprobleme GrippehnlicheSymptome Atemprobleme HerzKreislaufProbleme Temperregulierungsstrungen Sehstrungen |  |  |
| Muskel~e Gripp~me Atempr~e HerzKr~e Temper~n Sehstr~n |  |  |
| Muskelprob~e 1.0000 |  |  |
| Grippehnli~e 0.2515 1.0000 |  |  |
| Atemprobleme 0.3149 0.1540 1.0000 |  |  |
| HerzKreisl~e 0.2731 0.2118 0.3883 1.0000 |  |  |
| Temperregu~n 0.2824 0.2142 0.2987 0.3174 1.0000 |  |  |
| Sehstrungen 0.2007 0.1590 0.3794 0.2555 0.2366 1.0000 |  |  |
| . tetrachoric Muskelprobleme GrippehnlicheSymptome Atemprobleme HerzKreislaufProbleme Temperregulierungsstrungen Sehstrungen |  |  |
| (obs=512) |  |  |
| Muskel~e Gripp~me Atempr~e HerzKr~e Temper~n Sehstr~n |  |  |
| Muskelprob~e 1.0000 |  |  |
| Grippehnli~e 0.4506 1.0000 |  |  |
| Atemprobleme 0.5583 0.2626 1.0000 |  |  |
| HerzKreisl~e 0.4859 0.3720 0.6284 1.0000 |  |  |
| Temperregu~n 0.4979 0.3730 0.4945 0.5293 1.0000 |  |  |
| Sehstrungen 0.3719 0.2703 0.5693 0.4367 0.4018 1.0000 |  |  |
| . factor Muskelprobleme GrippehnlicheSymptome Atemprobleme HerzKreislaufProbleme Temperregulierungsstrungen Sehstrungen, factors(6) |  |  |
| (obs=512) |  |  |
| Factor analysis/correlation Number of obs = 512 |  |  |
| Method: principal factors Retained factors = 2 |  |  |
| Rotation: (unrotated) Number of params = 11 |  |  |
| -------------------------------------------------------------------------- |  |  |
| Factor Eigenvalue Difference Proportion Cumulative |  |  |
| -------------+------------------------------------------------------------ |  |  |
| Factor1 1.53617 1.44913 1.3461 1.3461 |  |  |
| Factor2 0.08704 0.14702 0.0763 1.4224 |  |  |
| Factor3 -0.05998 0.03215 -0.0526 1.3699 |  |  |
| Factor4 -0.09213 0.02024 -0.0807 1.2891 |  |  |
| Factor5 -0.11237 0.10521 -0.0985 1.1907 |  |  |
| Factor6 -0.21758 . -0.1907 1.0000 |  |  |
| -------------------------------------------------------------------------- |  |  |
| LR test: independent vs. saturated: chi2(15) = 393.53 Prob>chi2 = 0.0000 |  |  |
| Factor loadings (pattern matrix) and unique variances |  |  |
| ------------------------------------------------- |  |  |
| Variable Factor1 Factor2 Uniqueness |  |  |
| -------------+--------------------+-------------- |  |  |
| Muskelprob~e 0.4955 0.1151 0.7412 |  |  |
| Grippehnli~e 0.3627 0.1793 0.8363 |  |  |
| Atemprobleme 0.6041 -0.1357 0.6167 |  |  |
| HerzKreisl~e 0.5561 -0.0119 0.6906 |  |  |
| Temperregu~n 0.5078 0.0635 0.7382 |  |  |
| Sehstrungen 0.4764 -0.1381 0.7539 |  |  |
| ------------------------------------------------- |  |  |
| . rotate, varimax |  |  |
| Factor analysis/correlation Number of obs = 512 |  |  |
| Method: principal factors Retained factors = 2 |  |  |
| Rotation: orthogonal varimax (Kaiser off) Number of params = 11 |  |  |
| -------------------------------------------------------------------------- |  |  |
| Factor Variance Difference Proportion Cumulative |  |  |
| -------------+------------------------------------------------------------ |  |  |
| Factor1 1.16027 0.69733 1.0167 1.0167 |  |  |
| Factor2 0.46294 . 0.4057 1.4224 |  |  |
| -------------------------------------------------------------------------- |  |  |
| LR test: independent vs. saturated: chi2(15) = 393.53 Prob>chi2 = 0.0000 |  |  |
| Rotated factor loadings (pattern matrix) and unique variances |  |  |
| ------------------------------------------------- |  |  |
| Variable Factor1 Factor2 Uniqueness |  |  |
| -------------+--------------------+-------------- |  |  |
| Muskelprob~e 0.3679 0.3514 0.7412 |  |  |
| Grippehnli~e 0.2208 0.3391 0.8363 |  |  |
| Atemprobleme 0.5890 0.1909 0.6167 |  |  |
| HerzKreisl~e 0.4846 0.2730 0.6906 |  |  |
| Temperregu~n 0.4046 0.3133 0.7382 |  |  |
| Sehstrungen 0.4803 0.1238 0.7539 |  |  |
| ------------------------------------------------- |  |  |
| Factor rotation matrix |  |  |
| -------------------------------- |  |  |
| Factor1 Factor2 |  |  |
| -------------+------------------ |  |  |
| Factor1 0.8606 0.5093 |  |  |
| Factor2 -0.5093 0.8606 |  |  |
| -------------------------------- |  |  |
| . sem (Muskelprobleme GrippehnlicheSymptome Atemprobleme HerzKreislaufProbleme Temperregulierungsstrungen Sehstrungen <- Muskel), method(ml) | latent | (Muskel) |
| Endogenous variables |  |  |
| Measurement: Muskelprobleme GrippehnlicheSymptome Atemprobleme HerzKreislaufProbleme Temperregulierungsstrungen Sehstrungen |  |  |
| Exogenous variables |  |  |
| Latent: Muskel |  |  |
| Fitting target model: |  |  |
| Iteration 0: log likelihood = -1531.1226 |  |  |
| Iteration 1: log likelihood = -1530.3607 |  |  |
| Iteration 2: log likelihood = -1530.3475 |  |  |
| Iteration 3: log likelihood = -1530.3475 |  |  |
| Structural equation model Number of obs = 512 |  |  |
| Estimation method = ml |  |  |
| Log likelihood = -1530.3475 |  |  |
| ( 1) [Muskelprobleme]Muskel = 1 |  |  |
| OIM |  |  |
| Coef. Std. Err. z P>z [95% Conf. Interval] |  |  |
| Measurement |  |  |
| Muskelprobleme <- |  |  |
| Muskel 1 (constrained) |  |  |
| _cons .8515625 .0157125 54.20 0.000 .8207666 .8823584 |  |  |
| ------------------------------+---------------------------------------------------------------- |  |  |
| GrippehnlicheSymptome <- |  |  |
| Muskel .8304327 .1413677 5.87 0.000 .5533572 1.107508 |  |  |
| _cons .7675781 .0186666 41.12 0.000 .7309923 .804164 |  |  |
| ------------------------------+---------------------------------------------------------------- |  |  |
| Atemprobleme <- |  |  |
| Muskel 1.742606 .2170817 8.03 0.000 1.317133 2.168078 |  |  |
| _cons .6386719 .0212302 30.08 0.000 .5970614 .6802824 |  |  |
| ------------------------------+---------------------------------------------------------------- |  |  |
| HerzKreislaufProbleme <- |  |  |
| Muskel 1.313893 .1683017 7.81 0.000 .984028 1.643758 |  |  |
| _cons .8007813 .0176517 45.37 0.000 .7661845 .835378 |  |  |
| ------------------------------+---------------------------------------------------------------- |  |  |
| Temperregulierungsstrungen <- |  |  |
| Muskel 1.187156 .1578812 7.52 0.000 .8777141 1.496597 |  |  |
| _cons .7890625 .0180301 43.76 0.000 .7537242 .8244008 |  |  |
| ------------------------------+---------------------------------------------------------------- |  |  |
| Sehstrungen <- |  |  |
| Muskel 1.352514 .1928315 7.01 0.000 .9745712 1.730457 |  |  |
| _cons .6035156 .0216183 27.92 0.000 .5611445 .6458868 |  |  |
| Variance |  |  |
| e.Muskelprobleme .0950256 .006964 .0823113 .1097037 |  |  |
| e.GrippehnlicheSymptome .1567629 .0104589 .1375476 .1786626 |  |  |
| e.Atemprobleme .1354844 .0124074 .1132238 .1621216 |  |  |
| e.HerzKreislaufProbleme .1053618 .008495 .0899609 .1233993 |  |  |
| e.Temperregulierungsstrungen .1222202 .0091191 .1055926 .1414662 |  |  |
| e.Sehstrungen .1818844 .0132193 .1577357 .20973 |  |  |
| Muskel .0313783 .0063534 .0210999 .0466635 |  |  |
| LR test of model vs. saturated: chi2(9) = 20.78, Prob > chi2 = 0.0137 |  |  |
| . estat gof, stats(all) |  |  |
|  |  |  |
| Fit statistic Value Description |  |  |
| Likelihood ratio |  |  |
| chi2_ms(9) 20.780 model vs. saturated |  |  |
| p > chi2 0.014 |  |  |
| chi2_bs(15) 395.715 baseline vs. saturated |  |  |
| p > chi2 0.000 |  |  |
| Population error |  |  |
| RMSEA 0.051 Root mean squared error of approximation |  |  |
| 90% CI, lower bound 0.022 |  |  |
| upper bound 0.079 |  |  |
| pclose 0.442 Probability RMSEA <= 0.05 |  |  |
| Information criteria |  |  |
| AIC 3096.695 Akaike's information criterion |  |  |
| BIC 3172.985 Bayesian information criterion |  |  |
| Baseline comparison |  |  |
| CFI 0.969 Comparative fit index |  |  |
| TLI 0.948 Tucker-Lewis index |  |  |
| Size of residuals |  |  |
| SRMR 0.029 Standardized root mean squared residual |  |  |
| CD 0.703 Coefficient of determination |  |  |
|  |  |  |
| . estat mindices |  |  |
| Modification indices |  |  |
|  |  |  |
| Standard |  |  |
| MI df P>MI EPC EPC |  |  |
| Covariance |  |  |
| e.Muskelprobleme |  |  |
| e.GrippehnlicheSymptome 6.045 1 0.01 .0150231 .1230882 |  |  |
| --------------------------+----------------------------------------------- |  |  |
| e.GrippehnlicheSymptome |  |  |
| e.Atemprobleme 8.052 1 0.00 -.0235995 -.1619332 |  |  |
| --------------------------+----------------------------------------------- |  |  |
| e.Atemprobleme |  |  |
| e.Sehstrungen 10.135 1 0.00 .0320796 .2043559 |  |  |
| EPC = expected parameter change |  |  |
|  |  |  |
|  |  |  |
|  |  |  |

Results2, new

HAUPTANALYSE

dauer_kat

│

┌────────────┴────────────┐

│	│

Gruppe 1	Gruppe 2

≤ 2 Jahre	3–8 Jahre

│	│

│	interne Prüfung

│	der Stabilität

│	│

│	48 vs. 96 Monate

│	│

└────────────┬────────────┘

│

Gruppe 3

> 8 Jahre

SENSITIVITÄTSANALYSE

dauer4

│

≤2 | 3–10 | 11–20 | >20

│

Prüfung: verändert sich das Messmodell mit der Krankheitsdauer?

sem (Muskelprobleme GrippehnlicheSymptome Atemprobleme HerzKreislaufProbleme Temperregulierungsstrungen Sehstrungen <- muscle) if inlist(dauert_kat,1,2,3), group(dauert_kat) method(ml) latent(muscle) ginvariant(mcoef)

| Iteration 15999: log likelihood = -2118.8255 (not concave) |  |  |
| --- | --- | --- |
| Iteration 16000: log likelihood = -2118.8255 (not concave) |  |  |
| convergence not achieved |  |  |
| Structural equation model Number of obs = 736 |  |  |
| Grouping variable = dauer_kat Number of groups = 3 |  |  |
| Estimation method = ml |  |  |
| Log likelihood = -2118.8255 |  |  |
| ( 1) [Muskelprobleme]1bn.dauer_kat#c.muscle = 1 |  |  |
| ( 2) [GrippehnlicheSymptome]1bn.dauer_kat#c.muscle - [GrippehnlicheSymptome]3.dauer_kat#c.muscle = 0 |  |  |
| ( 3) [Atemprobleme]1bn.dauer_kat#c.muscle - [Atemprobleme]3.dauer_kat#c.muscle = 0 |  |  |
| ( 4) [HerzKreislaufProbleme]1bn.dauer_kat#c.muscle - [HerzKreislaufProbleme]3.dauer_kat#c.muscle = 0 |  |  |
| ( 5) [Temperregulierungsstrungen]1bn.dauer_kat#c.muscle - [Temperregulierungsstrungen]3.dauer_kat#c.muscle | = | 0 |
| ( 6) [Sehstrungen]1bn.dauer_kat#c.muscle - [Sehstrungen]3.dauer_kat#c.muscle = 0 |  |  |
| ( 7) [Muskelprobleme]2.dauer_kat#c.muscle = 1 |  |  |
| ( 8) [GrippehnlicheSymptome]2.dauer_kat#c.muscle - [GrippehnlicheSymptome]3.dauer_kat#c.muscle = 0 |  |  |
| ( 9) [Atemprobleme]2.dauer_kat#c.muscle - [Atemprobleme]3.dauer_kat#c.muscle = 0 |  |  |
| (10) [HerzKreislaufProbleme]2.dauer_kat#c.muscle - [HerzKreislaufProbleme]3.dauer_kat#c.muscle = 0 |  |  |
| (11) [Temperregulierungsstrungen]2.dauer_kat#c.muscle - [Temperregulierungsstrungen]3.dauer_kat#c.muscle = | 0 |  |
| (12) [Sehstrungen]2.dauer_kat#c.muscle - [Sehstrungen]3.dauer_kat#c.muscle = 0 |  |  |
| (13) [Muskelprobleme]3.dauer_kat#c.muscle = 1 |  |  |
| (14) [mean(muscle)]1bn.dauer_kat = 0 |  |  |
| OIM |  |  |
| Coef. Std. Err. z P>z [95% Conf. Interval] |  |  |
| Measurement |  |  |
| Muskelprobleme <- |  |  |
| muscle |  |  |
| [*] 1 (constrained) |  |  |
| _cons |  |  |
| =2 J .8582677 .0214745 39.97 0.000 .8161785 .900357 |  |  |
| 38 J .7327818 .0311681 23.51 0.000 .6716933 .7938702 |  |  |
| >8 J .736381 .0256354 28.73 0.000 .6861366 .7866255 |  |  |
| ------------------------------+---------------------------------------------------------------- |  |  |
| GrippehnlicheSymptome <- |  |  |
| muscle |  |  |
| [*] .7413253 .1314375 5.64 0.000 .4837124 .9989381 |  |  |
| _cons |  |  |
| =2 J .7204724 .028319 25.44 0.000 .6649682 .7759767 |  |  |
| 38 J .6802843 .0378504 17.97 0.000 .606099 .7544696 |  |  |
| >8 J .672181 .0304215 22.10 0.000 .6125559 .7318061 |  |  |
| ------------------------------+---------------------------------------------------------------- |  |  |
| Atemprobleme <- |  |  |
| muscle |  |  |
| [*] 1.87702 .2119338 8.86 0.000 1.461638 2.292403 |  |  |
| _cons |  |  |
| =2 J .7007874 .0292336 23.97 0.000 .6434905 .7580843 |  |  |
| 38 J .3580534 . . . . . |  |  |
| >8 J .3887797 . . . . . |  |  |
| ------------------------------+---------------------------------------------------------------- |  |  |
| HerzKreislaufProbleme <- |  |  |
| muscle |  |  |
| [*] 1.400505 .1596638 8.77 0.000 1.08757 1.713441 |  |  |
| _cons |  |  |
| =2 J .8110236 .0245281 33.07 0.000 .7629494 .8590978 |  |  |
| 38 J .6162988 .0372052 16.56 0.000 .5433778 .6892197 |  |  |
| >8 J .6238534 .0308303 20.24 0.000 .5634271 .6842797 |  |  |
| ------------------------------+---------------------------------------------------------------- |  |  |
| Temperregulierungsstrungen <- |  |  |
| muscle |  |  |
| [*] 1.293734 .1526213 8.48 0.000 .994602 1.592866 |  |  |
| _cons |  |  |
| =2 J .7834646 .0255952 30.61 0.000 .7332988 .8336303 |  |  |
| 38 J .5660074 .0398048 14.22 0.000 .4879915 .6440233 |  |  |
| >8 J .639419 .031047 20.60 0.000 .578568 .7002699 |  |  |
| ------------------------------+---------------------------------------------------------------- |  |  |
| Sehstrungen <- |  |  |
| muscle |  |  |
| [*] 1.443742 .182547 7.91 0.000 1.085957 1.801528 |  |  |
| _cons |  |  |
| =2 J .6062992 .0307831 19.70 0.000 .5459654 .666633 |  |  |
| 38 J .4077687 .0453812 8.99 0.000 .3188233 .4967142 |  |  |
| >8 J .4646839 .0355266 13.08 0.000 .3950529 .5343148 |  |  |
| Mean |  |  |
| muscle |  |  |
| =2 J 0 (constrained) |  |  |
| 38 J .1422182 .025443 5.59 0.000 .0923508 .1920857 |  |  |
| >8 J .1457846 .0216664 6.73 0.000 .1033192 .1882499 |  |  |
| Variance |  |  |
| e.Muskelprobleme |  |  |
| =2 J .0929175 .0092999 .0763664 .1130557 |  |  |
| 38 J .082022 .0101331 .0643831 .1044935 |  |  |
| >8 J .083021 .0074193 .0696818 .0989137 |  |  |
| e.GrippehnlicheSymptome |  |  |
| =2 J .1903913 .0173534 .1592441 .2276306 |  |  |
| 38 J .1605218 .0180574 .1287599 .2001186 |  |  |
| >8 J .1530028 .0127648 .1299227 .1801829 |  |  |
| e.Atemprobleme |  |  |
| =2 J .131753 .0157703 .1042018 .1665889 |  |  |
| 38 J .1333983 .019752 .0997963 .1783143 |  |  |
| >8 J .1347766 .0145421 .1090867 .1665165 |  |  |
| e.HerzKreislaufProbleme |  |  |
| =2 J .1053164 .0114578 .0850924 .1303472 |  |  |
| 38 J .0938702 .0128711 .0717487 .1228121 |  |  |
| >8 J .0969175 .0095387 .0799146 .117538 |  |  |
| e.Temperregulierungsstrungen |  |  |
| =2 J .1258687 .0129191 .1029322 .1539163 |  |  |
| 38 J .1356489 .0169447 .106191 .1732785 |  |  |
| >8 J .1067407 .0099141 .0889755 .1280531 |  |  |
| e.Sehstrungen |  |  |
| =2 J .1902155 .0188342 .1566621 .2309552 |  |  |
| 38 J .1890928 .0228942 .1491479 .2397359 |  |  |
| >8 J .1634384 .0149758 .136571 .1955913 |  |  |
| muscle |  |  |
| =2 J .0242157 .0052824 .0157913 .0371344 |  |  |
| 38 J .0275722 .0062383 .0176964 .0429595 |  |  |
| >8 J .0240702 .004815 .0162631 .035625 |  |  |
| Note: [*] identifies parameter estimates constrained to be equal across groups. |  |  |
| LR test of model vs. saturated: chi2(37) = 51.96, Prob > chi2 = 0.0523 |  |  |
| convergence not achieved |  |  |
| r(430); |  |  |
| . estat ginvariant |  |  |
| Tests for group invariance of parameters |  |  |
|  |  |  |
| Wald Test Score Test |  |  |
| chi2 df p>chi2 chi2 df p>chi2 |  |  |
| Measurement |  |  |
| Muskelprobleme <- |  |  |
| muscle . . . 1.860 2 0.3946 |  |  |
| _cons 15.524 2 0.0004 . . . |  |  |
| ------------------------------+----------------------------------------------------------------- |  |  |
| GrippehnlicheSymptome <- |  |  |
| muscle . . . 3.285 2 0.1935 |  |  |
| _cons 1.390 2 0.4990 . . . |  |  |
| ------------------------------+----------------------------------------------------------------- |  |  |
| Atemprobleme <- |  |  |
| muscle . . . 1.401 2 0.4964 |  |  |
| _cons 137.451 1 0.0000 . . . |  |  |
| ------------------------------+----------------------------------------------------------------- |  |  |
| HerzKreislaufProbleme <- |  |  |
| muscle . . . 0.252 2 0.8817 |  |  |
| _cons 26.718 2 0.0000 . . . |  |  |
| ------------------------------+----------------------------------------------------------------- |  |  |
| Temperregulierungsstrungen <- |  |  |
| muscle . . . 1.500 2 0.4723 |  |  |
| _cons 22.600 2 0.0000 . . . |  |  |
| ------------------------------+----------------------------------------------------------------- |  |  |
| Sehstrungen <- |  |  |
| muscle . . . 1.272 2 0.5295 |  |  |
| _cons 14.585 2 0.0007 . . . |  |  |
| Mean |  |  |
| muscle . . . . . . |  |  |
| Variance |  |  |
| e.Muskelprobleme 0.903 2 0.6366 . . . |  |  |
| e.GrippehnlicheSymptome 3.119 2 0.2102 . . . |  |  |
| e.Atemprobleme 0.022 2 0.9889 . . . |  |  |
| e.HerzKreislaufProbleme 0.545 2 0.7616 . . . |  |  |
| e.Temperregulierungsstrungen 2.847 2 0.2409 . . . |  |  |
| e.Sehstrungen 1.665 2 0.4350 . . . |  |  |
| muscle 0.498 2 0.7794 . . . |  |  |
| more-- |  |  |

Results, new
